# Hydromechanical Modulation of Enzymatic Kinetics Using Microfluidically Configurable Nanoconfinement Arrays

**DOI:** 10.1021/acscentsci.4c01094

**Published:** 2024-10-21

**Authors:** Yunjie Wen, Yutao Li, Henry C. W. Chu, Shibo Cheng, Yong Zeng

**Affiliations:** † Department of Chemistry, University of Florida, Gainesville, Florida 32611, United States; ‡ Department of Chemical Engineering, University of Florida, Gainesville, Florida 32611, United States; § Department of Mechanical and Aerospace Engineering, University of Florida, Gainesville, Florida 32611, United States; ∥ J. Crayton Pruitt Family Department of Biomedical Engineering, University of Florida, Gainesville, Florida 32611, United States; ⊥ University of Florida Health Cancer Center, Gainesville, Florida 32611, United States

## Abstract

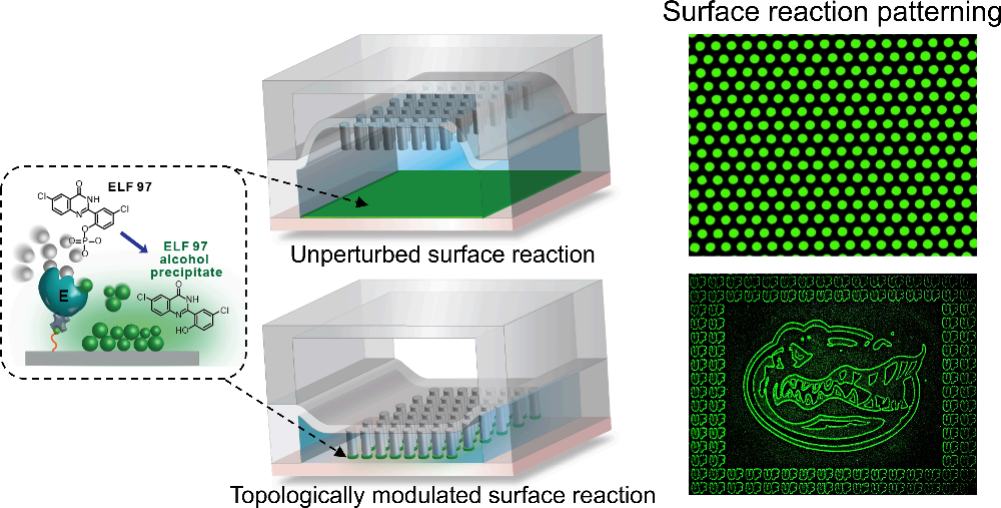

Confinement of molecules occurs ubiquitously in nature and fundamentally affects their properties and reactions. Developing synthetic confinement systems capable of precise modulation of chemical reactions is critical to understanding the underlying mechanisms and to promoting numerous applications including biosensing. However, current nanoconfinement systems often require sophisticated fabrication and operation. Here we report a simplified nanoconfinement approach termed **C**onfigurable **H**ydromechanical **E**nzyme **M**odulation by **N**anoconfinement **L**andscaping **o**f **C**hemical **K**inetics (CHEMNLOCK). This approach exploits a simple micropost device to generate an array of nanogaps with tunable geometries, enabling flexible spatial modulation of the kinetics of surface-bound enzymatic reactions and substantial enhancement of single-molecule reactions. We envision that the CHEMNLOCK concept could pave a new way for developing scalable and practical nanoconfinement systems with profound impacts on biosensing and clinical diagnostics.

## Abstract 2

A configurable nanoconfinement system was built upon pneumatically actuatable microfluidic devices to enable hydromechanical modulation and patterning of surface enzymatic reactions.

## Introduction

Confinement of molecules occurs ubiquitously in nature and fundamentally affects their properties and associated reactions. In living systems, confinements are considered instrumental in mediating biological processes, including stabilization, storage, transportation, interactions, and synthesis of biomolecules.^1,2^ Such confinement effects have been shown to result in enhanced kinetics,^3^ extraordinary selectivity,^4^ and precise control^5^ of chemical reactions. For instance, surface-bound or compartmentalized enzymes within cells manage a complex network of biochemical reactions in an efficient, timely, spatially organized, and physiologically optimal manner.^6^ Inspirations from nature have drawn extensive interest in exploring artificial nanoconfinement systems that imitate biological conditions to deliver these appealing features.^7,8^ These synthetic systems promise to address current challenges in a broad range of fields, including catalysis, energy, biosensing, and pharmaceutics.^7,9^

Prevalent confining strategies encompass volumetric encapsulation using molecular^10^ or physical compartmentalization and surface/interface immobilization of molecules on solid supports. Recent remarkable advances in nanomaterials and nanotechnology provide a myriad of promising platforms, such as nanoparticles,^11^ nanochannels,^12^ 2D materials,^13^ and nanoporous structures,^14^ to develop synthetic confining systems. These nanoscale materials and devices offer unique properties, such as ultrahigh surface-to-volume ratio and the ability to manipulate the spatial distribution and/or orientation of enzymes,^15^ to control the thermodynamics and kinetics of confined reactions.^7,9^ It was observed that nanoconfinements result in the acceleration of biochemical reactions,^6,16^ improved catalytic activities of enzymes,^17,18^ favorable shift in reaction equilibrium of antibody–antigen binding,^12,19^ and enhancement or alteration of selectivity.^6,7^ Developing new systems that precisely modulate nanoconfinement effects is essential to elucidating the principles governing confinement-modulated reactivity, which will shed new insights in complex biological processes and promote their broad applications.

An increasingly growing application of nanoconfinements is to develop new biosensing platforms. Various forms of nanoconfinements have been explored for biosensing, including nanoporous materials (e.g., graphene,^20,21^ metal–organic frameworks,^22,23^ and nanogels^24,25^), nanocapsules,^26,27^ and nanofabricated devices.^28,29^ These nanoscale systems affect the biosensing processes via many factors, including large surface area, increased local concentration of reactants, promoted mass transfer, and enhanced physical interactions and molecular binding between the target and sensing agents, leading to the improved analytical sensitivity, specificity, and speed. Multilength-scale engineering has attracted growing interests in biosensing as this strategy marries unique micro- and nanoscale phenomena to immensely improve existing biosensors and develop new sensing mechanisms.^30^ In addition to nanomaterials, micro/nanofabricated systems offer an effective means to create precisely defined artificial nanoconfinements. Such devices permit greater control over the key factors that influence reaction equilibrium and kinetics in target binding, amplification, and/or detection. Moreover, these micro/nanochip systems are inherently amenable to the integration of an analytical workflow to build fully integrated biosensing devices. Despite these advantages, there are major challenges in the broad adaptation of these nanodevices to real-world applications. Standard nanofabrication suffers from sophisticated facilities, time-consuming and costly procedures, and limited scalability. Technical challenges can also arise in reproducible operation of nanofabricated devices which requires specialized control instruments and extensive sample processing to mitigate the risk of clogging and surface fouling.

**Figure 1:**
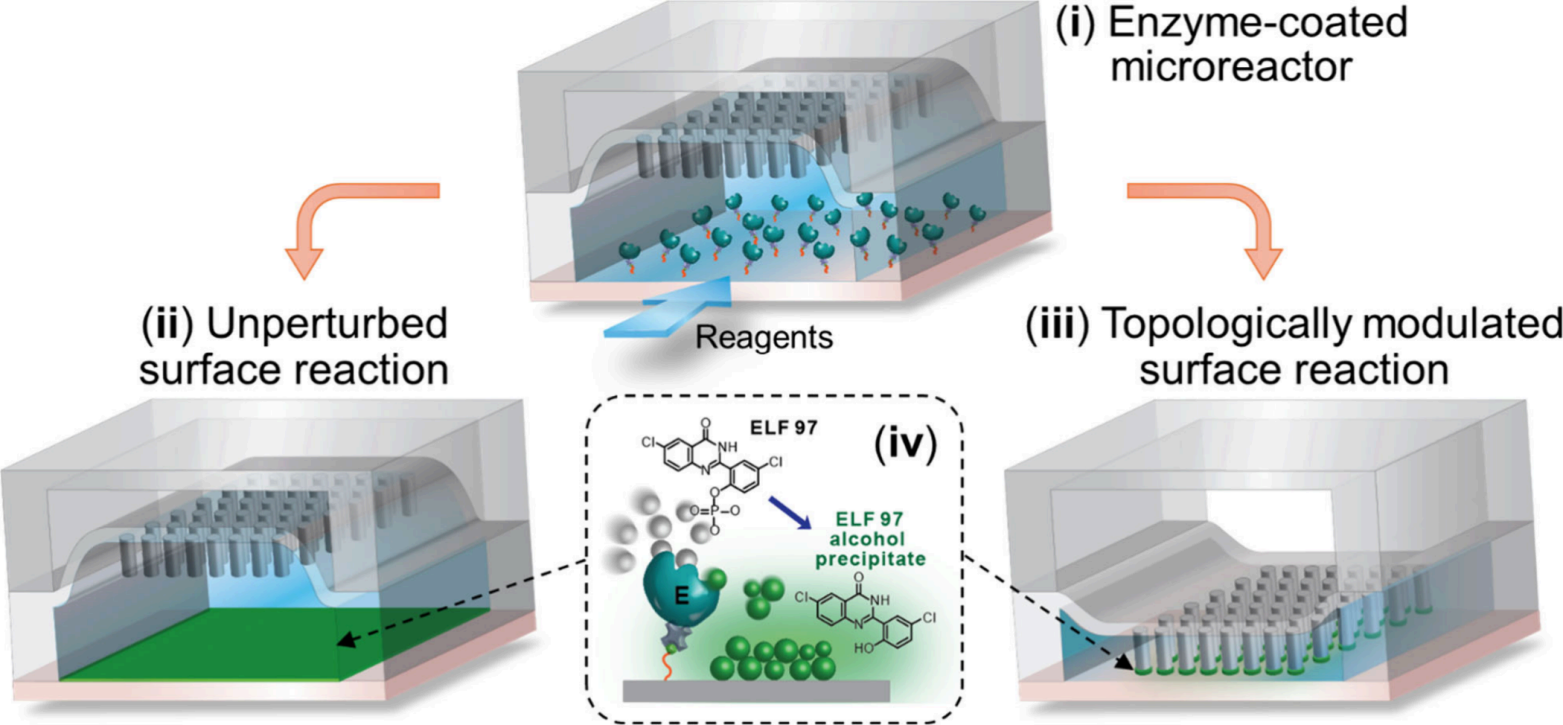
Schematic of CHEMNLOCK. Conceptual illustration of the CHEMNLOCK strategy that modulates and enhances surface enzymatic reactions. Surface enzymatic reactions in the enzyme-coated microreactor (i) can be conducted in the unperturbed mode (ii) or in the modulation mode (iii). Alkaline phosphatase (ALP)/ELF-97 (iv) as the enzyme/substrate pair is investigated in this study.

Herein we developed a simple and robust method that affords configurable mechanical modulation of surface enzymatic reaction termed **C**onfigurable **H**ydromechanical **E**nzyme **M**odulation by **N**anoconfinement **L**andscaping **o**f **C**hemical **K**inetics (CHEMNLOCK). Built on our previous study,^31^ the CHEMNLOCK system exploits a pneumatically actuatable micropost array to enable hydromechanical formation of nanogaps between the microposts and the glass substrate with adjustable geometries (Figure 1). We conducted both numerical simulations and experimental investigations to examine the mechanism and capability of CHEMNLOCK for enzyme modulation in nanogaps. We demonstrated that CHEMNLOCK affords spatial modulation of enzyme reactivity on a surface via controlling the balance between mass transfer and the reaction kinetics, enabling programmable landscaping of surface-bound enzymatic catalysis. Therefore, CHEMNLOCK presents a new strategy that repurposes a well-developed simple microdevice as an effective nanoconfinement system to enable configurable engineering and patterning of surface enzymatic reactions in a noncontact manner, as well as to promote its potential applications in biosensing and clinical medicine.

## Results and Discussion

### CHEMNLOCK Exploits Micropost Arrays to Pattern Well-Defined Nanoconfinements

The CHEMNLOCK approach was inspired by our previous observation that a thin film of aqueous solution will be trapped by pneumatically pressing a polydimethylsiloxane (PDMS) microstructure onto a hydrophilic glass surface and the film thickness can be tuned by varying the actuation pressure.^31^ We hypothesize that this phenomenon can be harvested to create configurable confinements to spatially modulate and enhance enzymatic reactions on a planar surface. To test this hypothesis, we investigated a model system in which a pneumatically actuatable microreactor was used to perturb the enzymatic activity of alkaline phosphatase (ALP) immobilized on the substrate surface, as conceptually illustrated in Figure 1.

The device has a three-layer PDMS/glass construct in which the middle PDMS membrane is patterned by a micropost array with the same height as the flow channel and the glass surface of the reaction chamber is coated uniformly by ALP. The micropost array can be lifted by vacuum to quickly fill the microreactor with a solution of ALP substrate (Figure 1, (i)). For comparison, surface enzymatic reaction can be performed in an unperturbed mode with the post array held up (Figure 1, (ii)) or in the modulation mode with the post array pressed down at variable pressures (Figure 1, (iii)). In both cases, the fluid flow in the microreactor is stopped to prevent hydrodynamic disturbance of the spatial distribution of the enzymatic reaction products. For this proof-of-principle study, we choose a soluble, nonfluorescent substrate, ELF-97, which can be hydrolyzed by ALP into insoluble, fluorescent ELF-97 alcohol that precipitates out (Figure 1, (iv)). This ALP/ELF-97 reaction provides a well-poised model for our study because its precipitate product is (1) tightly localized to the site of enzymatic activity for activity detection with superior spatial resolution,^32^ and (2) photostable and strongly fluorescent for reliable and sensitive signal detection.^33^ These unique characteristics permit convenient visualization of the micropost-modulated enzymatic activity landscape with good sensitivity and resolution using standard fluorescence microscopy imaging.

### CHEMNLOCK Chip

**Figure 2:**
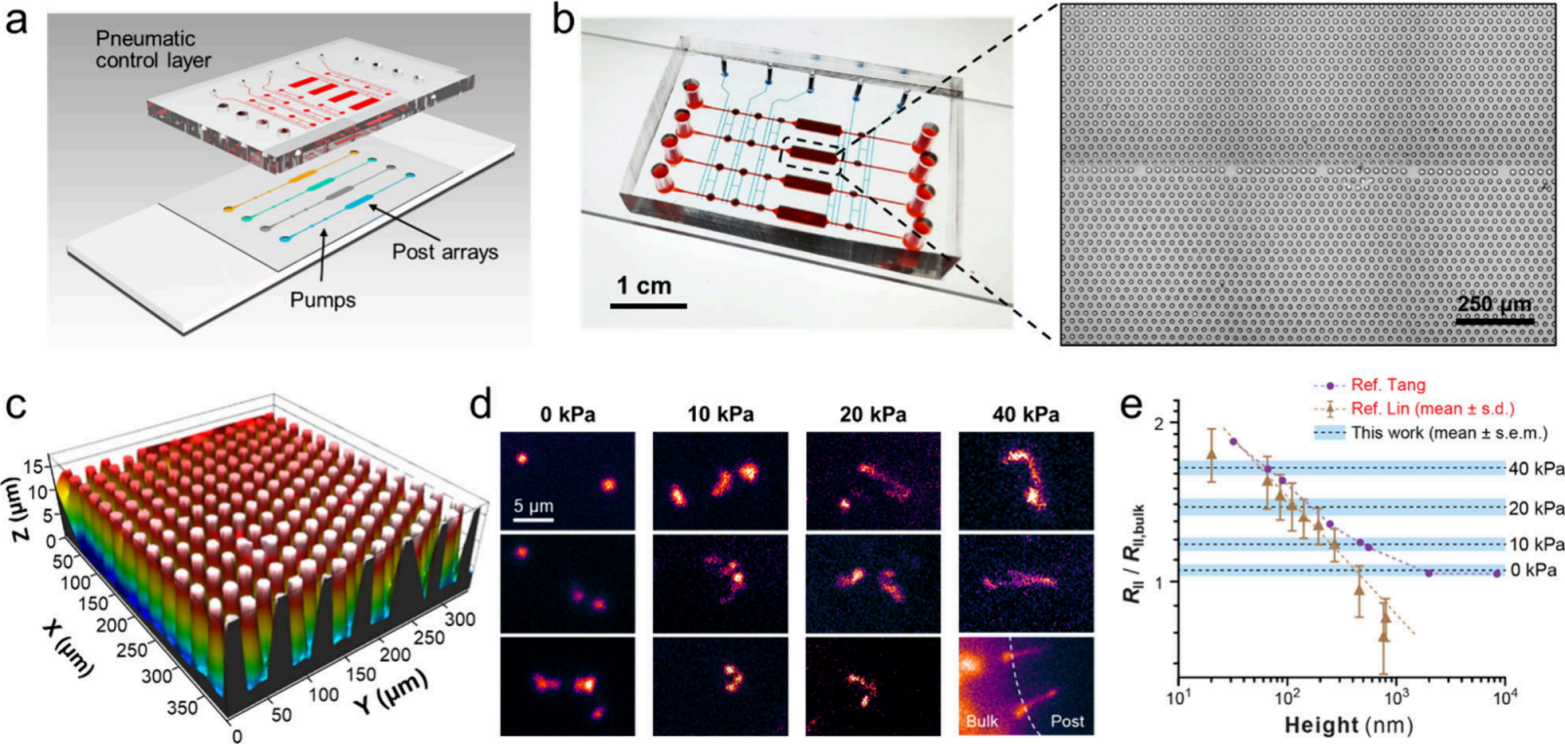
Fabrication and characterization of the CHEMNLOCK device. (a) Design of the CHEMNLOCK chip composed of a pneumatic control circuit and an array of four parallel microreactors patterned with the micropost arrays. (b) Digital photo of a CHEMNLOCK chip showing the microreactor array with the micropost structures (magnified image). (c) Optical profilometry plot of the array of 15-μm microposts. (d) Snapshots of λ-DNA molecules confined by microposts of 80-μm diameter at pressing pressures of 0, 10, 20, and 40 kPa. (e) Scaled in-plane radius of gyration of λ-DNA (*R*_||_/*R*_||,bulk_) determined at different pressing pressures. Reference plots of *R*_||_/*R*_||,bulk_ as a function of slit height reported from Tang et al.^34^ and Lin et al.^35^ are also included.

We designed a PDMS/glass hybrid chip composed of a pneumatic control circuit and an array of four parallel microreactors patterned with microposts (Figure 2a). Each microreactor is flanked by a 3-valve micropump for precise control of reagent delivery and a lifting gate microvalve for stopping the fluid flow for enzymatic reaction.^36^ The devices were microfabricated using a multilayer soft lithography process^37,38^ detailed in Methods. Figure 2b displays a completed microchip with ∼15-μm tall flow channels and the microposts of 15-μm diameter fabricated on a ∼150-μm thick PDMS layer. As visualized by the noncontact optical profilometry (Figure 2c), the fabricated microposts show a conical frustum shape with a slightly reduced top diameter of 11.6 ± 0.9 μm, which is owing to the nonuniform UV exposure across a thick photoresist film resulting in the lithographic structures with nonvertical sidewalls. The diameter and spacing distance of the microposts varied from 10 to 160 μm, as specified below.

As mentioned previously, our approach was inspired by the observation that a thin layer of aqueous solution can be formed by pneumatically pressing a PDMS microstructure onto a hydrophilic glass surface under relatively small pressures.^31,39^ Compared to solid thin films, accurate thickness measurement of transparent liquid film at the nanometer scale remains a technical challenge under extensive investigations and mostly relies on optical methods that require highly sophisticated instruments and careful calibration against a reliable reference.^40,41^ A simple method based on fluorescence imaging of spatially confined single DNA molecules provides a more accessible means for convenient estimation of the dimensions of nanofluidic structures.^34,35,42^ Therefore, we adopted this approach to characterize the slit-like gap created between a micropost and the substrate by visualizing the conformational changes of individual λ-DNA molecules in relation to the characteristic confining dimension which is the gap height (*H*). The full contour length and the bulk radius of gyration (*R*_g,bulk_) of 48.5 kb λ-DNA stained with the YOYO-1 dye in a good solvent have been experimentally measured to be in the range of ∼18–25 μm and ∼0.7–1 μm, respectively, depending on the measurement methods, dye to base pair ratio, and solvent conditions.^34,35,43−47^Figure 2d shows typical images of YOYO-1-labeled λ-DNA (dye to base pair ratio of 1:6) confined by the microposts of 80-μm diameter at different pressing pressures. Most of the λ-DNA molecules observed at 0 kPa resembled free-solution DNA in globular random coil conformation with only slight deformation. As the pressing pressure was elevated, λ-DNA became more anisotropically extended; and at 40 kPa linear chains were commonly seen, whose length can reach >50% of the full contour length of λ-DNA (Figure 2d). Such changes in λ-DNA conformation agree qualitatively with the transition from the weak confinement when *H* ∼ 2*R*_g,bulk_ to the moderate (Kuhn length *L*_K_ < *H* < *R*_g*,*bulk_) and strong confinements (*H* < *L*_K_) that was observed in the fabricated nanoslits with a height ranging from ∼30 nm to 2 μm.^34,43^

For more quantitative assessment of the micropost confinement, we measured the averaged in-plane radius of gyration for λ-DNA floating in the microchannel (*R*_||,bulk_) and confined under the microposts (*R*_||_) as described before (molecule number *n* > 50 for each condition).^35,45^ The measured *R*_||,bulk_ (0.80 ± 0.11 μm) for λ-DNA in 1× TE buffer is in line with the values reported with the same labeling ratio and similar TE buffers.^45,46^Figure 2e presents the scaled in-plane radius of gyration of λ-DNA (*R*_||_/*R*_||, bulk_) determined at different pressures and the reference plots of *R*_||_/*R*_||,bulk_ as a function of slit height reported from two independent studies.^34,35^ Considering the observed weak slit confinement of λ-DNA and the reference plot covering a broad range of nanoslit height (∼32 nm to 8.5 μm),^34^ we estimated the gap height created at 0 kPa (*H*_0 kPa_) to be within ∼1.3–2 μm. Given the quantitative discrepancy between two reference plots in the moderate confinement regime (de Gennes regime), our estimate of the slit height at 10 kPa falls in a range of *H*_10 kPa_ = ∼270–600 nm, which is much larger than the typical Kuhn length for dsDNA (*L*_K_ = ∼100 nm). The nanogap confinement formed at 20 and 40 kPa appears to transition into the Odijk regime where the two reference plots agree well, allowing us to extract their height estimates to be *H*_20 kPa_ = ∼100–190 nm and *H*_40 kPa_ = ∼ 50–80 nm. These *H* estimates also agree well with the values extracted from other relevant studies carried out with various experimental and simulation conditions.^43,44,48^ We note that despite its simplicity and convenience, the DNA imaging method yields semiquantitative measurement of the confining geometry. More accurate and precise characterization of the nanogap formation in our device requires systematic investigation using the sophisticated optical methods for liquid thin film analysis, which is beyond the scope of this exploratory proof-of-concept study.

### Mechanistic Studies of CHEMNLOCK

**Figure 3:**
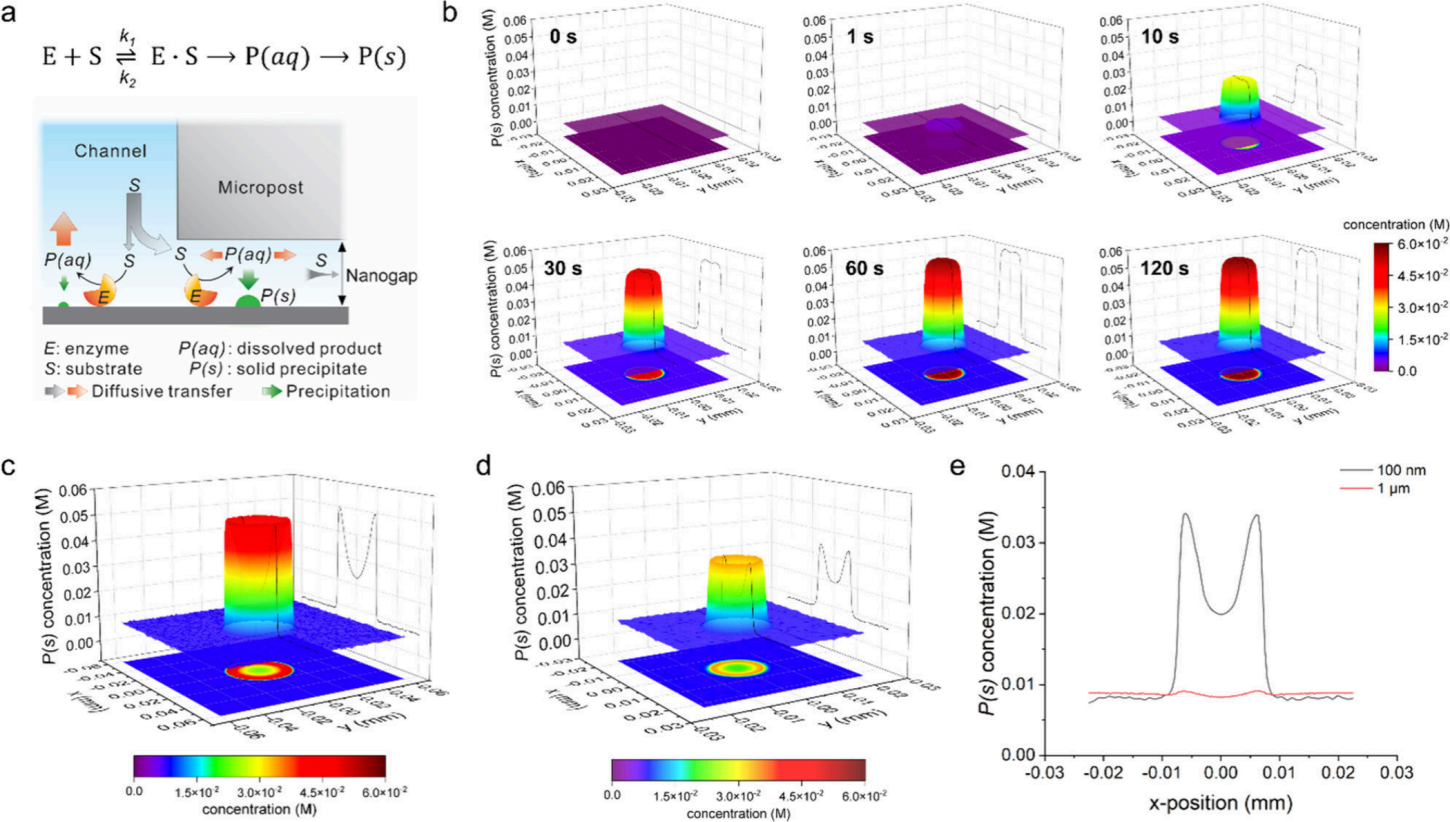
Mechanistic studies of CHEMNLOCK by numerical simulation. (a) Proposed enzyme kinetics model of ALP/ELF-97 reaction (top) and the schematic of reaction processes and mass transport processes modulated by the micropost-defined nanogap (bottom). Four species are involved in the system: enzyme (*E*), substrate (*S*), dissolved product (*P*(aq)), and solid product (*P*(s)). The diffusive transport of *S* (gray arrow) and *P*(aq) (orange arrow) and the precipitation of *P*(aq) (green arrow) are highlighted. The thickness of colored arrows represents the proceeding extent of a specific process. The thicker the arrow is, the more extent the process proceeds to. (b) Simulation results showing the time evolution of the surface concentration profile of *P*(s). Post diameter (*d*) = 15 μm. Simulation rate constants: *k*_1_ = 10^–6^ m/s, *k*_2_ = 10^–8^ m/s, and *k*_p_ = 10^3^ s^–1^. The sectional concentration profile at *y* = 0 mm and the projection of the surface concentration profile are also displayed. Color contours indicate the concentration magnitude. (c) Simulation results showing the surface concentration profile of *P*(s) at *t* = 120 s with *d* increased to 40 μm. Simulation rate constants: *k*_1_ = 10^–6^ m/s, *k*_2_ = 10^–8^ m/s, and *k*_p_ = 10^3^ s^–1^. The sectional concentration profile at *y* = 0 mm and the projection of the surface concentration profile are also displayed. Color contours indicate the concentration magnitude. (d) Simulation results showing the surface concentration profile of *P*(s) at *t* = 120 s with *k*_1_ increased to 10^–5^ m/s. *d* = 15 μm. *k*_2_ = 10^–8^ m/s. *k*_p_ = 10^3^ s^–1^. The sectional concentration profile at *y* = 0 mm and the projection of the surface concentration profile are also displayed. Color contours indicate the concentration magnitude. (e) Simulation results comparing the enhancement of *P(s)* concentration using different gap heights. Concentration profiles are taken at *y* = 0 mm and *t* = 120 s. *d* = 15 μm. Simulation rate constants: *k*_1_ = 10^–5^ m/s, *k*_2_ = 10^–8^ m/s, and *k*_p_ = 10^3^ s^–1^.

To facilitate the mechanistic study of the CHEMNLOCK process, we first conducted numerical simulations of the micropost-induced perturbation of surface ALP/ELF-97 reaction using a simplified enzyme kinetic model (see Methods and Supporting Information, SI, for the simulation details). This model couples a classic enzyme kinetics equation with a step of product precipitation which is assumed to be irreversible owing to the very low solubility and fast precipitation of ELF-97 alcohol^49^ (Figure 3a, top). In our case, the enzyme (*E*) is uniformly distributed on the bottom surface of the microreactor. The substrate (*S*) in the microchannel diffuses to the surface to react with the enzyme, producing the dissolved product (*P*(aq)) near the surface that will diffuse into the bulk solution (Figure 3a, bottom). If the local concentration of *P*(aq) accumulates to reach the saturation level, it will precipitate to form the solid product, *P(s)*. Submicron-scale confinements have been shown to enhance surface-bound enzyme reactions and affinity binding.^6,12,50,51^ Thus, we hypothesize that compared to the open channel region, the surface enzymatic reaction confined under a micropost can be enhanced, creating a stronger concentration gradient of *S* to drive its preferential diffusive transport toward the confined area to sustain the fast reaction. This effect accelerates the production of *P*(aq) within the nanogap to reach the saturation level and precipitate out. The precipitating process will be further expedited in the nanogap as the micropost restricts the vertical diffusion of *P*(aq) into the bulk to boost its local concentration near the surface. Because the micropost also restricts the mass transport of *S* from the bulk into the nanogap, the overall confinement effect is determined by the dynamic competition between the surface reaction and the replenishment of *S* via lateral diffusion along the radius of the micropost. At the sites near the micropost edge with low spatial impedance on diffusion, the reaction can be enhanced and maintained due to the large flux of *S*. As the travel distance toward the center increases, more *S* will be consumed, transitioning the kinetics-limited surface reaction to a diffusion-limited process. If the enzymatic reaction is very fast, then significant depletion of *S* can outcompete the confinement-induced enhancement and even suppress the reaction in the inner area of the nanogap. Using a configurable micropost device, our method can control the mass transport to enable topological modulation of the reaction kinetics and patterning of the reaction products on a surface.

To expedite the computing process, our modeling is focused on the kinetic interplay between mass transport and surface reaction, ignoring other possible molecular-scale factors that may contribute to the enhanced enzyme reactivity under nanoconfinement, including surface charge, conformational change of immobilized proteins, and shift of reaction equilibrium.^6,9,12,50−52^ Given the fact that gap height used here (≥100 nm) is much larger than the calculated Debye length (<1 nm) and the reported dimensions of ALP (∼10 nm × 5 nm × 5 nm for *E. coli* ALP),^53^ such simplification is reasonable and should afford conservative assessment of the surface enzyme kinetics in a multilength-scale confining system without the need for excessive computational efforts and time. The reaction rates in our model were characterized by a set of first-order rate constants^49,54−58^ and detailed in the SI. We first simulated the time evolution of the surface enzymatic reaction in a single-post nanogap system (15 μm in diameter and 100 nm in height, SI Figure S1). The simulation results show that compared to the open channel surface, the nanogap confinement enhances the reaction rate to reach the saturation level of *P*(aq) (SI Figure S2) and significantly elevates the production of *P*(s) within the nanogap (Figure 3b). Moreover, the simulated concentration profile of *S* displays a stronger concentration gradient to drive preferential transport of *S* from the bulk space to the nanogap versus the open bottom surface (SI Figure S3), sustaining the accelerated reaction in the nanoconfinement. Simulation of different multipost systems yielded the consistent behavior when the ratio of post diameter to post interval was kept the same (SI Figure S4). These results qualitatively capture the kinetics picture predicted by the model (Figure 3a) and support the important impacts of micropost confinement on the dynamic interplay between the surface enzyme kinetics and mass transport as described above.

We further assessed the nanogap confinement effects by adjusting the simulation variables to probe the processes of surface enzymatic reaction and mass transport. First, we adjusted the nanogap geometry that directly regulates the mass transport in our confinement system. It is expected that increasing the nanogap radius will restrict mass transport of *S* to the center and weaken the enhancement effect. Indeed, the simulation with a micropost of 40 μm in diameter showed that the enhancement effect was peaking near the rim of the nanogap and decaying toward the center (Figure 3c), indicating the transition of the kinetics-limited surface reaction to a diffusion-limited process.

We then tuned the surface reaction kinetics by varying the reaction rate constants. The simulation showed that a relative high reaction rate (e.g., *k*_1_ = 10^–5^ m/s) can lead to reduced enhancement and even notable suppression of the reaction at the central area of the nanogap (SI Figure S5a). This can be attributed to the sufficiently fast consumption of *S* at the open channel surface that depletes the inward supply of *S*, resulting in a transition from the reaction-limited to diffusion-limited kinetics along the micropost’s radius. In contrast, lowering the reaction rate, i.e., reducing *k*_1_ to 10^–6^ m/s and 10^–7^ m/s, resulted in stronger reaction enhancement at the center of the nanogap (SI Figure S5b and S5c), indicating the dominance of diffusive transport of *S* over the surface consumption of *S* within the nanogap.

For the 15-μm micropost device, suppression of the reaction enhancement also occurred when the forward reaction rate constant was increased by 10 folds (Figure 3d). This demonstrates the advantage of smaller confining elements to afford consistent surface reaction enhancement over a broad range of reaction kinetics. The nanogap height is another important dimension in modulating the nanoconfinement effect as it affects mass transport in both radial and vertical direction. We conducted the simulation comparing two gap heights of 100 nm and 1 μm formed with a 15-μm micropost. As seen in Figure 3e, the nanoconfinement-induced enhancement of the surface enzymatic reaction was almost completely diminished when the gap height was increased to 1 μm. As reasoned previously (Figure 3a), this result can be attributed to the combination of two effects: (1) reduced enhancement of surface enzyme reactivity under weaker confinement^6,50−52^ and (2) slower precipitation process because the produced *P*(aq) near the surface can diffuse away more easily with less vertical spatial restriction. In sum, these results together predict the ability of our micropost-based confining strategy to modulate surface enzymatic reactions via tuning the enzyme kinetics and mass transport.

### Surface Reaction Modulation by CHEMNLOCK

**Figure 4:**
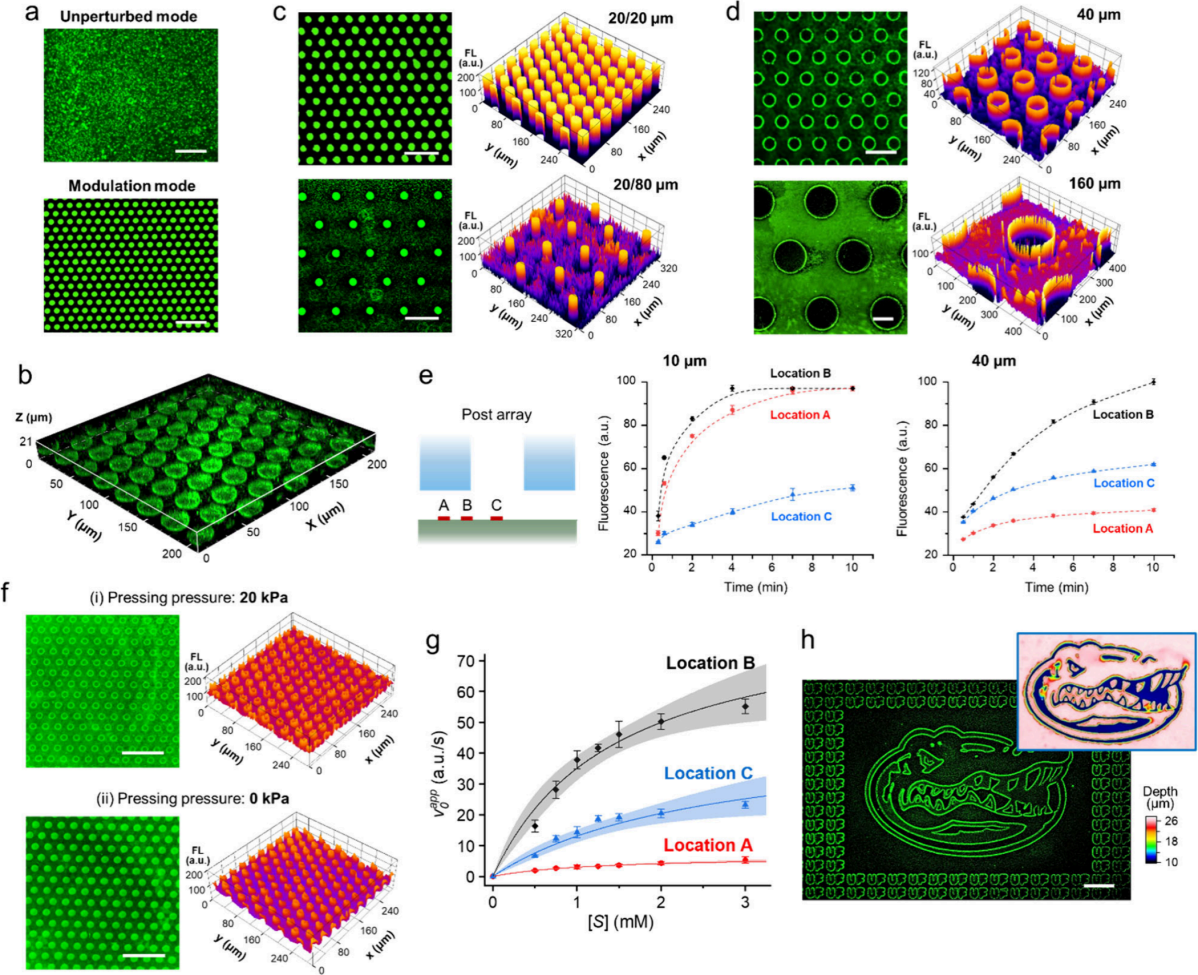
Hydromechanical modulation of surface enzymatic reaction by CHEMNLOCK. (a) Comparisons of ALP/ELF-97 reaction conducted in the unperturbed mode (top) or the modulation mode (bottom). Post diameter (*d*) = post spacing (*l*) = 15 μm. (b) 3D confocal fluorescence microscopy image showing the spatial distribution of ELF-97 alcohol precipitates inside the microreactor. *d* = *l* = 15 μm. (c) Left, representative fluorescence microscopy images of ALP/ELF-97 reaction conducted using post array designs (*d*/*l*) of 20/20 μm (top) and 20/80 μm (bottom). Right, surface plots of the images showing the fluorescence intensity. (d) Left, fluorescence images of ALP/ELF-97 reaction conducted using post array designs (*d*/*l*) of 40/40 μm (top) and 160/160 μm (bottom). Right, surface plots of the images showing the fluorescence intensity. (e) Time-lapse plots of the fluorescence intensity at three designated locations (left) using 10-μm (middle) and 40-μm (right) posts. Error bars represent one SD (*n* = 3). (f) Effects of reaction kinetics and mass transport on the nanoconfined surface reaction. (i) Fluorescence images of ALP/ELF-97 reaction using 2.5 mM ELF-97 and 20 kPa pressing pressure. (ii) Fluorescence images of ALP/ELF-97 reaction using 2.5 mM ELF-97 and 0 kPa pressing pressure. Surface plots of the images showing the fluorescence intensity are also displayed. *d* = *l* = 15 μm. (g) Michaelis–Menten curves fitted from the apparent initial reaction rates measured at the three designated locations as a function of substrate concentration. Shadow areas indicate 95% confidence bands of the fitting curves. Error bars represent one SD (*n* = 5). (h) Fluorescence image of the pattern of UF hallmarks and Florida Gators printed by contactless spatial modulation of the enzymatic production of ELF-97 alcohol precipitates. Inset, optical profilometry plot of the Florida Gators pattern in a positive stamp fabricated by photolithography. Color contours indicate the depth magnitude. All scale bars: 100 μm.

We experimentally assessed the modulation of surface ALP/ELF-97 reaction enabled by the CHEMNLOCK strategy (see Methods). The device surface was blocked to minimize the nonspecific adsorption of enzyme molecules. Using an array of posts with the 15-μm diameter and 15-μm spacing, we first compared the reaction (1.2 μg/mL streptavidin conjugated ALP and 0.5 mM ELF-97) conducted with the post array lifted (the unperturbed mode) or pressed down (the modulation mode), as illustrated in Figure 4a. As expected, the unperturbed enzymatic reaction led to uniform surface distribution of the fluorescent ELF-97 alcohol precipitates. On the contrary, with the microposts pressed down, the enzymatic production of the precipitates was greatly enhanced under the entire confined regions, while the reaction on the unmasked surface was suppressed (Figure 4a). Using confocal fluorescence microscopy, we observed that the ELF-97 alcohol precipitates were mostly generated underneath the microposts, especially near the edge of microposts, rather than the open channel surface or the side walls of the microposts (Figure 4b). This observation agrees qualitatively with the behavior of the nanogap-modulated surface reaction predicted by our model (Figure 3b), which confirms the micropost-defined nanoconfinement as the dominant factor to induce the noncontact patterning of surface reaction.

We further investigated these nanoconfinement effects under the same assay conditions via varying the geometrical design of the micropost array. The microposts with a diameter increased to 20 μm could also effectively enhance the ALP/ELF-97 reaction across the confined area underneath (Figure 4c). Same as that seen in Figure 4a, the reaction on the open channel surface was largely suppressed in the micropost array with the 20-μm spacing (Figure 4c, top), indicating the overlapped depletion zones formed around microposts due to preferential transport of the substrate to the confined regions (SI Figure S3). This was verified by the observation of the separate depletion zones around individual microposts when the spacing was increased to 80 μm (Figure 4c, bottom). When the post diameter was increased to 40 μm or larger with the same diameter/spacing ratio, a donut-shaped distribution of the fluorescent product was displayed, indicating the intensified enzymatic reaction around the rim of the nanogap and the suppressed reaction in the middle area (Figure 4d). We quantitatively characterized the micropost-confined reaction kinetics by monitoring the real-time fluorescence signals at three surface locations in a micropost array: (A) the center of a post, (B) the inner point that is ∼2 μm from the post edge, and (C) the middle point between two adjacent posts (Figure 4e, left). It is possible that some precipitates were deposited on the bottom surface of the microposts because the enzyme and substrate concentrations used here were high. These precipitates were also detected for characterizing the confined enzymatic kinetics, as they are a part of the product of the confined enzymatic reactions. For an array of 10-μm posts, the average signals measured at the locations A and B increase at a rate enhanced by ∼2.4 folds and ∼2.2 folds of that at the location C, respectively, over the first 2 min reaction time (Figure 4e, middle). While increasing at a slightly lower rate, the signal levels at the center location A eventually approached that at the location B, indicating the confined reaction being dominantly governed by the surface reaction kinetics rather than the diffusive transport of the substrate and reaction products. On the contrary, for an array of 40-μm posts, the signal level at the location A was reduced drastically and lower than that at the location C (Figure 4e, right). Such post size-dependent change in the surface reaction landscape matches nicely with our simulation results (Figure 3b, c), which manifests the spatial transition from the reaction-limited to the mass transport-limited enzymatic kinetics inward along the radius of a nanogap.

We then investigated the effects of reaction kinetics and mass transport on the nanoconfined surface reaction by adjusting the experimental variables that govern the reaction rate and spatial confinement, respectively. As depicted in Figure 4f (i), when the ELF-97 concentration was increased from 0.5 to 2.5 mM, the donut-shaped patterning of the surface ALP/ELF-97 reaction could also be obtained with smaller microposts, such as the 15-μm microposts. This observation is in line with that of our simulation studies (Figure 3d, SI Figure S5) where the surface reaction was expedited to suppress the diffusive transport of the substrate toward and thus the reaction in the central area of the nanogaps. Enlarging the height of nanogap can promote the mass transfer to enhance the reaction in the center of nanogaps. As expected, when we raised the 15-μm microposts from <200 nm to ∼1–2 μm in height by reducing the pressing pressure from 20 to 0 kPa (Figure 2e), more uniform enhancement of the reaction across the nanogaps was achieved even at the fast reaction rate, which is shown in Figure 4f (ii). However, the weaker confinement generated with the lower pressing pressure resulted in less local enhancement of the surface enzymatic reaction, consistent with the theoretical prediction on the effect of nanogap height on the CHEMNLOCK process (Figure 3e).

The nanoconfinement-modulated ALP/ELF-97 reaction kinetics was systematically evaluated with the Michaelis–Menten model. In this case, the enzymatic assays were conducted with an array of 40-μm microposts for which 1.2 μg/mL streptavidin conjugated ALP was used for surface coating and the ELF-97 concentration varied from 0.5 to 3 mM. We measured the formation of fluorescent precipitates for 10 min at the above-mentioned three surface locations (see Methods). The apparent initial rates, *v*_0_^app^, were plotted against the substrate concentration, [*S*], and the apparent Michaelis–Menten parameters, *K*_M_^app^ and *V*_max_^app^, were obtained from the fitting curves, as shown in Figure 4g and SI Table S2. The key steady-state assumption () for Michaelis–Menten model holds validity in our study given the linearity of the rate of formation of fluorescent precipitates with respect to the time window of our measurement (SI Figure S6). Enzymes exhibited decreasing *K*_M_^app^ from location C to location A, indicating an increasing affinity to the substrates from the open bottom surface to the nanogap center. The improvement of enzyme–substrate affinity can be attributed to the restricted diffusion of the pre-existed substrates within the confined space which allows more interactions with the enzymes relative to the open channel surface.^6,9,50^ The slightly larger *K*_M_^app^ at location B than at location A can further verify this point as there is a lower spatial impedance on diffusion at the sites near the micropost edge compared to the center of the nanogap. In the meantime, enzymes at location B showed the largest *V*_max_^app^ which is ∼12.7-fold and ∼1.8-fold that at locations A and C, respectively. *V*_max_^app^ comprehensively describes the formation of fluorescent precipitates including the conversion of substrates into dissolved products, the precipitation of dissolved products, and the mass transfer of all reaction species. The significant increase in *V*_max_^app^ at location B manifests the nanoconfinement effects to expedite the enzymatic reaction, burst the precipitation of ELF-97 alcohol, as well as sustain the fast reaction via the preferential diffusive transport of substrates into the nanogap. Meanwhile, the magnificent decrease of *V*_max_^app^ at location A suggests a reduced enzymatic reactivity at the nanogap center due to the mass transport-limited kinetics. By further comparing the value of  which indicates the overall enzymatic efficiency at these three locations, we discover that our approach brings up the best enzymatic performance near the edge of microposts (location B), followed by the open channel surface (location C) and the nanogap center (location A). The results agree well with our prediction of the mechanisms underlying the nanoconfinement effects on surface enzymatic reactions (Figure 3a) and demonstrate the capability of our approach to regulate surface enzymatic reactivity and modulate the reactions.

Overall, these experimental findings verify the micropost-induced modulation of the surface enzymatic reaction which enhances the reaction kinetics, promotes the mass transfer of substrate to the nanogap areas, and thus depletes the substrate supply to the unconfined surface reaction. For a deeper investigation into the potential of our CHEMNLOCK method, we demonstrated the high-resolution, contactless printing of complex patterns on glass substrates by modulating the ALP/ELF-97 reaction with a microfabricated positive stamp of the University of Florida (UF) hallmarks and Florida Gators (Figure 4h). The contours of UF hallmarks and Florida Gators were highlighted by the enhanced production of ELF-97 alcohol precipitates. Collectively, our studies suggest that the CHEMNLOCK strategy affords flexible configurability to modulate the surface enzymatic reaction simply by tunning the nanogap geometry.

### Modulation of Surface-Bound Single-Molecule Reactions

**Figure 5:**
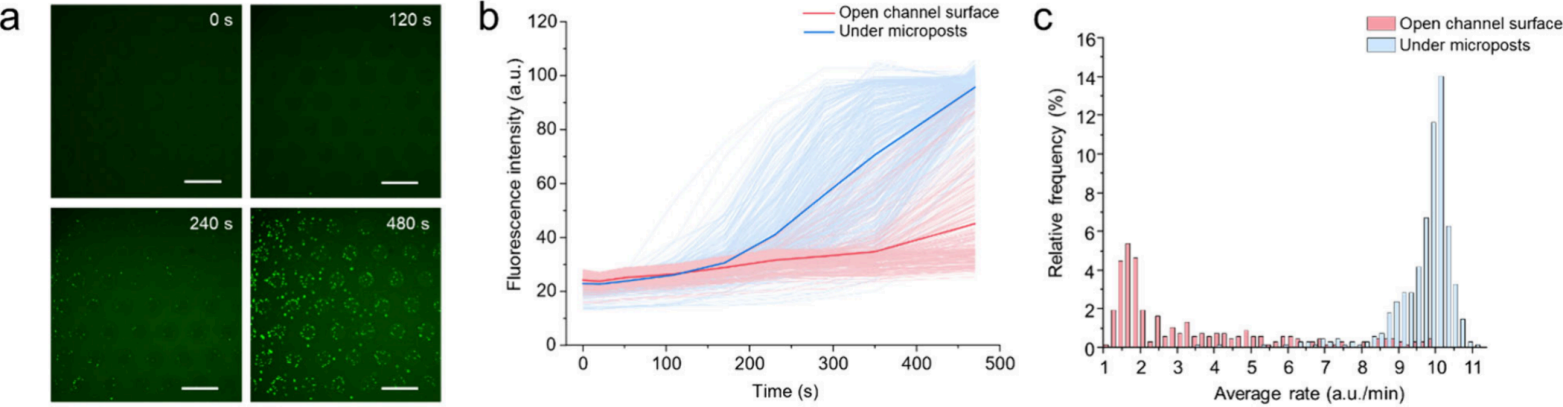
Controlling single-molecule enzymatic reactions by CHEMNLOCK. (a) Representative fluorescence images showing the time evolution of CHEMNLOCK-enhanced slow ALP/ELF-97 reaction. Scale bars: 100 μm. (b) Typical time courses of the intensity of fluorescent dots at different locations. Highlighted blue and pink represent the time evolution of the average intensity of all individual fluorescent dots under microposts and at open channel surface, respectively. Light blue and pink represent the time evolution of the intensity of all individual fluorescent dots under microposts and at open channel surface, respectively. (c) Histogram showing the comparison of average reaction rates at different locations.

To further explore the capacity of our CHEMNLOCK method, we decreased the concentration of the enzyme and substrate to investigate the CHEMNLOCK enhancement effects on slow reaction kinetics. Unlike the donut-shaped distribution of fluorescent products under fast reaction kinetics, discrete distribution of individual fluorescent dots was observed with time (Figure 5a and SI Figure S7) when a slow reaction setup (0.6 μg/mL streptavidin conjugated ALP and 250 μM ELF-97) was applied. Such unique phenomenon can be attributed to the decreased surface density of enzymes as well as less overlap and merge among fluorescent aggregates due to decreased substrate concentration. Most fluorescent dots were formed under the microposts while only a small amount of them were found in the open channel area. We measured the fluorescence intensity change of these individual dots over the first 8 min of the reaction (Figure 5b). The time courses showed two distinctive populations: low-activity ones on the open channel surface (Figure 5b, light pink) and high-activity ones under microposts (Figure 5b, light blue). The average intensity of these two populations did not differ too much at the beginning of the reaction. While the open channel surface signals slightly increased over time, the ones under microposts showed a drastic increase after 2 min (Figure 5b, highlighted pink and blue). We then calculated the average reaction rate over the first 8 min for all fluorescent dots (Figure 5c). The histogram showed a clear separation of the reaction rates on the open channel surface and under microposts. The reaction rate under microposts is approximately 2.8-fold that on the open channel surface. Such results clearly demonstrate the capability of CHEMNLOCK to boost slow surface enzymatic reactions. Combined with the unique formation of individual fluorescent dots, our CHEMNLOCK approach provides the opportunity for enhanced single-molecule detection, paving the way for potential applications in digital bioassays.

### Modulation of HRP/TSA Reaction by CHEMNLOCK

**Figure 6:**
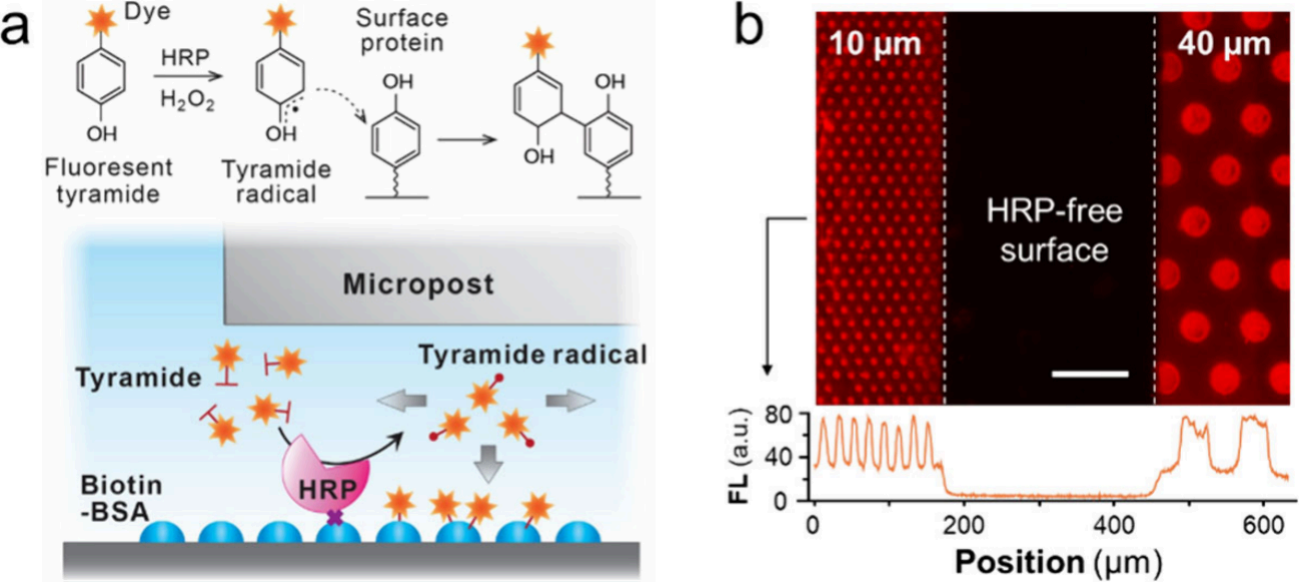
Adaptation of CHEMNLOCK to surface patterning of a different enzymatic reaction. (a) Top, principles of horseradish peroxide (HRP)/tyramide reaction and the deposition of dye-labeled tyramide substrate on a surface protein. Bottom, schematic of the CHEMNLOCK-modulated HRP/tyramide reaction. (b) Top, representative fluorescence microscopy image of HRP/tyramide reaction conducted using a mixture array of 10-μm and 40-μm posts. Bottom, fluorescence intensity profile of the image is displayed for the position indicated by the arrow. Scale bar: 100 μm.

As demonstrated above, the CHEMNLOCK strategy affords a simple and configurable mechanical approach to engineer biochemical reactions. In addition to the ALP/ELF-97 reaction, we also adapted the CHEMNLOCK strategy to modulate the horseradish peroxidase (HRP)/tyramide reaction, which is known as tyramide signal amplification (TSA) or catalyzed reporter deposition (CARD)^59^ (Figure 6a). Different from the heterogeneous process which involves the precipitation of fluorescent enzymatic products, HRP catalyzes the formation of the fluorescent dye-labeled tyramide radicals in the presence of hydrogen peroxide. The short-lived radicals will form covalent bonds with phenol residues on nearby proteins, depositing the fluorescent dye at the site of enzymatic generation. TSA also presents a well-poised signal detection modality as it permits high-density in situ labeling and sensitive visualization of enzymatic activity landscapes as the extremely short lifespan of tyramide radicals limits their diffusion distance upon generation to tens of nm.^60−62^ Here, we used a mixture array of 40- and 10-μm posts for the enzymatic assays for which 2 μg/mL streptavidin conjugated HRP was applied for surface coating followed by reaction with 10× fluorescent dye-labeled tyramide (see Methods). As shown in Figure 6b, the fluorescent dye-labeled tyramide formed clear boundaries between the HRP-coated and HRP-free surface region. We observed the enhancement of fluorescence signals under both 40- and 10-μm posts compared with those at the unconfined regions. Such enhancement can be attributed to similar mechanisms underlying the enhancement of ALP/ELF-97 reaction, but specifically for this case, the nanoconfinement enables more tyramide radicals to deposit within their lifespan via restricting the vertical diffusion of tyramide radicals and providing more binding sites compared with the unconfined area. These results demonstrate the potential of our approach as a more universal strategy to modulate and enhance surface biochemical reactions via simple and configurable mechanical designs.

## Conclusions

We introduced a new nanoconfinement strategy named CHEMNLOCK for configurable modulation of surface enzymatic reactions. A pneumatically actuatable micropost array was constructed to enable hydromechanical formation of nanogaps between the microposts and the glass substrate with adjustable geometries. Through modulating the interplay between mass transfer and the reaction kinetics, CHEMNLOCK affords programmable landscaping of enzyme reactivity at both ensemble and single-molecule scales. We observed either enhanced or suppressed enzymatic reactions which can be easily tuned by post geometry and pressing pressure, and demonstrated high-resolution, contactless printing of complex patterns with reaction products. We also demonstrated substantial enhancement of ultraslow reaction kinetics in this compartment-free nanoconfinement system with distinct dot-shaped products that can be potentially served for single-molecule counting in digital biosensing. Finally, our results demonstrated that the simple CHEMNLOCK method is applicable to various enzymatic reactions and thus may provide a broadly adaptable platform to enhance the performance of enzymatic amplification in different biosensing systems.

Compared to the existing methods, the CHEMNLOCK method demonstrated here presents some major advantages: (1) it exploits only simple microfluidic structures to afford configurable formation of nanoscale confinement, substantially promoting the reliability and scalability of device fabrication and operation; (2) this on-demand micronanofluidics-convertible mechanism eases direct implementation of various bioassays for analysis of complex samples with minimal pretreatment; and (3) its inherent compatibility with standard microfluidic engineering could facilitate the development of fully integrated and multiplexed biosensing microsystems. While the PDMS material offers appreciable advantages, such as low cost, ease of fabrication, and biocompatibility, our CHEMNLOCK system can also be limited by some properties of PDMS. For instance, compared to inorganic glass and silicon substrates, PDMS polymerization results in larger surface roughness and variation in elastomeric properties, which may limit the size control, uniformity, and reproducibility for the nanoconfinement formation by CHEMNLOCK. In addition, PDMS is known for its strong nonspecific adsorption of chemicals and biomolecules, which presents a common problem for potential applications. While a variety of methods have been previously reported to effectively suppress the nonspecific adsorption on PDMS surface, adaptation of the CHEMNLOCK system to specific applications would require addition efforts to optimize the conditions for surface treatment and the nanoconfined enzymatic reactions. Overall, our method could pave a distinct way for developing simple, scalable, and practically viable nanoconfinement technologies to promote their broad applications in basic research and clinical medicine, such as the development of novel digital immunoassays for disease diagnostics.

## Methods

### Reagents and Materials

Biotin-labeled bovine serum albumin (BSA) was purchased from Sigma–Aldrich. Carboxyethylsilanetriol (disodium salt, 25% in water), *N*-hydroxysuccinimide (NHS), 1-ethyl-3-(3-(dimethylamino)propyl) carbodiimide (EDC) hydrochloride, Blocker BSA (10% in PBS), λ DNA, YOYO-1 iodide, dithiothreitol (DTT), and ELF-97 (ex/em: 345/530) were obtained from Thermo Fisher Scientific. Streptavidin conjugated alkaline phosphatase (SA-ALP) was purchased from R&D Systems. Tyramide amplification kit with HRP streptavidin and CF640R dye tyramide was purchased from Biotium. 1× PBS and 1× TE buffers were obtained from Thermo Fisher Scientific and Integrated DNA Technologies, respectively. All other solutions were prepared with deionized water (18.2 MΩ·cm; Thermo Fisher Scientific). ALP and ELF-97 were prepared in 1× PBS which contains 25 mM Tris (Thermo Fisher Scientific), 10 mM MgCl_2_ (Sigma–Aldrich), and 1% BSA (Thermo Fisher Scientific) (ALP working buffer, pH 7.4). HRP and CF640R dye tyramide were prepared in PBS working solution (PBSW, pH 7.4) containing 1% BSA and tyramide amplification buffer provided by the manufacturer, respectively.

### Numerical Simulation

A computational species transport simulation was conducted using COMSOL Multiphysics to solve diffusion-reaction equations coupled with surface reactions through a finite-element approach. A simplified 3D geometry containing a single post that has identical dimensions to the experimental setups was used to model the micropost-induced perturbation of surface ALP/ELF-97 reaction (SI Figure S1). Diffusion coefficients of ELF-97 and dissolved ELF-97 alcohol molecules in PBS were estimated using Wilke-Chang correlation equations^63^ and are summarized in SI Table S1. A total of 53 850 and 339 867 elements were used for 3D 15-μm and 40-μm post design, respectively. A total of 23 571 and 65 143 elements were used for 2D 15-μm and 40-μm post design, respectively. Simulation equations and parameters can be found in SI.

### Microfabrication of Polydimethylsiloxane (PDMS) Chips

Two-layer PDMS chips were fabricated by multilayer soft lithography according to our established protocol.^37,38^ Briefly, silicon wafers were cleaned with piranha solution and spin-coated with SU-8 photoresist (MicroChem). For the mold of fluidic layer, 15-μm thick SU-8 2010 was spin-coated. For the molds of pneumatic layer and surface patterning chip, 50-μm and 30-μm thick SU-8 2025 were spin-coated, respectively. The SU-8 microstructures were fabricated onto the wafers from the photomasks, following the protocols recommended by the manufacturer. Prior to use, the SU-8 molds were treated with trichloro(1H,1H,2H,2H-perfluorooctyl) silane under vacuum overnight. To fabricate the pneumatic layer, 35 g mixture of PDMS base and curing agent at a 10:1 ratio was poured on the mold and cured in the oven at 70 °C for 4 h. The PDMS slabs were peeled off from the mold, cut, and punched to make pneumatic connection holes. Meanwhile, the fluidic layer was prepared by spin-coating the mold with 5 g mixture of PDMS base and curing agent at a ratio of 10:1 at 500 rpm for 30 s, followed by 700 rpm for 30 s. It was then cured in the oven at 70 °C for 4 h. To assemble the pneumatic layer and fluidic layer, they were treated by UV-Ozone for 5 min and manually aligned together under a stereomicroscope and permanently bonded by baking in the oven at 70 °C overnight. The two-layer PDMS slabs were then peeled off from the mold and reservoirs were punched.

### Measurement of Nanogap Heights with Fluorescence Imaging

The heights (*H*) of the slit-like gap at different actuation pressures were estimated via the fluorescence imaging of the conformational changes of individual λ-DNA molecules confined in the gap. λ-DNAs were first stained with YOYO-1 at a ratio of dye to base pair of 1:6 in 1× TE buffer (pH 8.0) containing 30 mM DTT at room temperature for 30 min. The λ-DNA solution was then 1:20 diluted in 1× TE buffer (pH 8.0). Microposts of 80 μm in diameter were chosen for the estimation of gap height. The CHEMNLOCK chip was first blocked with 5% BSA for 1.5 h, followed by washing with PBST, ddH_2_O, and 1× TE buffer (pH 8.0) sequentially. The diluted λ DNA solution was pumped in quickly to fill the chamber. The posts were then pressed down at different pressures (0, 10, 20, and 40 kPa). Valves on both sides were closed after pressing down the posts and the system was let stay for 5 min before imaging. The λ-DNA solution was repumped into the chamber each time for measurement at a new pressure. Imaging was performed using Zeiss Axio A1 fluorescence microscope with a 40× objective. The size of confined λ-DNA molecules was estimated by fitting them to homogeneous ellipses using ImageJ (NIH, http://rsbweb.nih.gov/ij/). Information of the radii of the fitting ellipse was obtained to calculate the average in-plane radius of gyration for λ-DNA molecules floating in the microchannel (*R*_||,bulk_) and confined under the posts (*R*_||_). Both *R*_||_ and *R*_||,bulk_ are given by , with *R*_M_ and *R*_m_ the radii of the ellipse along major and minor axes, respectively. *H* is then extracted by comparing the scaled in-plane radius of gyration of λ-DNA (*R*_||_/*R*_||, bulk_) determined at different pressures and the reference plots of *R*_||_/*R*_||,bulk_ as a function of slit height reported from two independent studies.^34,35^

### Modulation of Surface Enzymatic Reaction by CHEMNLOCK

The CHEMNLOCK chip was surface functionalized via EDC/NHS reaction for protein/antibody conjugation. Briefly, the glass slide was precleaned by piranha solution and treated with carboxyethylsilanetriol for 4 h. The glass slide was then washed with ddH_2_O and treated with EDC/NHS solution (2.3 mg/mL NHS and 2 mg/mL EDC) for 1 h. After washing with ddH_2_O, a patterning chip was assembled onto the glass slide and the solution of capture antibody/protein was flowed through the chip to coat the glass surface for 1 h at room temperature. The chip was then stored at 4 °C before the experiments. After removing the patterning chip, the surface-modified glass slide was dried by N_2_. The two-layer PDMS flow-channel chip was treated by UV-Ozone for 5 min and was aligned and assembled onto the glass slide to construct the complete CHEMNLOCK chip. 500 μg/mL biotinylated BSA was used as the capture protein to coat the surface. The micropost array was lifted by vacuum to allow the reagents to flow through in each step. Solutions were pneumatically pumped through the channel in a “stop-flow” manner.^64^ The CHEMNLOCK chips were first blocked with 5% BSA for 1.5 h. Streptavidin-conjugated ALP was prepared by 1:500 dilution in ALP working buffer. Ten μL of diluted ALP (1.2 μg/mL) was then pumped through the channel and reacted for 0.5 h. After washing away unbounded enzymes with 30 μL PBST, 5 μL 500 μM ELF-97 in ALP working buffer was quickly pumped into the chamber in 20 s. After the chamber was filled with ELF-97, the micropost array was pressed down at 20 kPa, followed by closing the flanking valves to stop the fluid flow. The reaction was performed for 0.5 h and then fluorescence images were taken using a Nikon Eclipse Ti2 inverted fluorescence microscope with a 20× objective.

Microposts of 40 and 10 μm in diameter were used to compare the reaction kinetics under different micropost sizes by monitoring the real-time fluorescence signals. After pressing down the microposts at 20 kPa (*t* = 0 s), fluorescence images were obtained at *t* = 20 s, 40 s, 2, 4, 5, 7, and 10 min. Background subtracted fluorescence intensity was measured at the three designated surface locations: (A) the center of a post, (B) the inner point that is 2 μm from the post edge, and (C) the middle point between two adjacent posts to make the time-lapsed plots using ImageJ.

### 3D Confocal Fluorescence Imaging

Confocal images were taken using a Nikon A1R MP Confocal/Multiphoton/STORM Microscope equipped with 405, 445, 488, 514, 561, and 647 nm solid-state lasers. A 60× long working distance oil objective was used. The laser intensity was 20% and the exposure time was 100 ms. Image stacks were taken at 0.5-μm interval along the *z*-axis ranging from the bottom of the glass substrate to the top of the pillar. The obtained image stacks were fitted into 3D view photography.

### Enzyme Kinetics Study Using the Michaelis–Menten Model

For experiments of enzyme kinetics study, a micropost array of 40 μm in diameter was used. We evaluated ELF-97 of different concentrations (500 μM, 750 μM, 1 mM, 1.25 mM, 1.5 mM, 2 mM, and 3 mM) with 1.2 μg/mL ALP. After the chamber is quickly filled with ELF-97, the micropost array was pressed down at 20 kPa. The moment when the micropost array was fully pressed down was manually picked as time zero point. The fluorescence images were taken every 2 s for 3 min followed by every 20 s for 7 min without moving the chip or camera view. Digital images were processed using ImageJ to measure the fluorescence intensity at the above-mentioned three surface locations. Five microposts were picked randomly to obtain the average fluorescence intensity. After obtaining the time-lapse plots, the apparent initial rate *v*_0_^app^ was calculated by linear fitting the first five points for each substrate concentration. *v*_0_^app^ was then plotted against the substate concentration to fit into the Michaelis–Menten model and the apparent Michaelis–Menten parameters, *K*_M_^app^ and *V*_max_^app^, were obtained from the fitting curves.

### Enhancement of Slow Surface Enzymatic Reaction by CHEMNLOCK

Microposts of 40 μm in diameter were used to monitor the real-time fluorescence signals. 0.6 μg/mL streptavidin conjugated ALP and 250 μM ELF-97 were used. Other steps were similar to the previous ALP/ELF-97 reaction setup. After pressing down the microposts at 20 kPa (*t* = 0 s), fluorescence images were obtained at *t* = 10 s, 30 s, 1, 2, 3, 4, 5, 6, 8, 10, 15, 20, 30, and 50 min. Fluorescence intensity of individual dots was measured using ImageJ. The average rate as the *x*-axis in Figure 5c was calculated as the fluorescence intensity increment of each dot in 8 min.

### TSA Reaction Modulated by CHEMNLOCK

A micropost mixture array of 40 and 10 μm in diameter was used. Ten μL of 2 μg/mL streptavidin conjugated HRP was injected and reacted for 1 h following blocking with 5% BSA for 1.5 h. After washing with 30 μL PBST, 5 μL 1× CF640R dye tyramide was quickly pumped into the chamber in 20 s and the micropost array was pressed down at 20 kPa after the chamber was filled with the dye tyramides. The reaction went for 0.5 h at room temperature. The micropost array was then lifted and 30 μL PBST was used to wash away the remaining dye tyramides. The micropost array was pressed down again at 20 kPa for fluorescence imaging.

### Statistical Analysis

Mean and standard deviation (S.D.) were calculated with standard formulas. All statistical analyses were conducted at a 95% confidence level using Excel 2018, OriginPro 2019, and GraphPad Prism 8.

## Data Availability

The authors declare that all data supporting the findings of this study are available within the paper and its Supporting Information. The raw and analyzed data sets generated during the study are available for research purposes from the corresponding author on reasonable request.

## Supporting Information Available

Simulation models and equations (Figure S1); addition simulation results (Figure S2–S5); time-lapse plots of the fluorescence intensity measured at the three designated locations using different ELF-97 concentrations (Figure S6); time evolution of CHEMNLOCK-enhanced slow ALP/ELF-97 reaction (Figure S7); summary of simulation constants (Table S1); and summary of the apparent Michaelis–Menten parameters for the enzymatic studies (Table S2) (PDF)
