# Supplementary material for: Hydromechanical Modulation of Enzymatic Kinetics Using Microfluidically Configurable Nanoconfinement Arrays
Source: ACS Cent Sci. 2024 Oct 21;10(11):2059–71. doi: 10.1021/acscentsci.4c01094 (PMC11613295; doi:10.1021/acscentsci.4c01094)
Supplement: Supplementary file 1 — oc4c01094_si_001.pdf [file oc4c01094_si_001.pdf]

Supplementary Information

Hydromechanical Modulation of Enzymatic Kinetics Using Microfluidically Configurable Nanoconfinement Arrays

Yunjie Wen[a,†], Yutao Li[a,†], Henry C. W. Chu[b,c], Shibo Cheng[a], and Yong Zeng[a,d,e,*]

[a] Department of Chemistry, University of Florida, Gainesville, FL 32611;
[b] Department of Chemical Engineering, University of Florida, Gainesville, FL 32611;
[c] Department of Mechanical and Aerospace Engineering, University of Florida, Gainesville, FL 32611;
[d] J. Crayton Pruitt Family Department of Biomedical Engineering, University of Florida, Gainesville, FL 32611;
[e] University of Florida Health Cancer Center, Gainesville, FL 32611;
[†] These authors contribute equally;
* Corresponding author: zengy@ufl.edu (Y. Zeng).

This file includes:
Figure S1. Simulation models and equations.
Figure S2-S5. Additional simulation results.
Figure S6. Time-lapse plots of the fluorescence intensity measured at the three designated locations using different ELF-97 concentrations.
Figure S7. Time evolution of CHEMNLOCK-enhanced slow ALP/ELF-97 reaction.
Table S1. Summary of simulation constants.
Table S2. Summary of the apparent Michaelis-Menten parameters for the enzymatic studies.

Simulation model

Geometry

The geometry of the simulation model of the micropost-induced perturbation of surface enzymatic reaction is shown in **Figure S1**. We consider a 3D cuboid microreactor with a single cylindrical post (**Figure S1, a**). The post is represented by the boundaries created by removing a cylinder from the cuboid. The post is at the center of the microreactor and has a height of h and a diameter of d. The microreactor has a depth and width of $3d$, and a height of $H + h$. The distance between the bottom boundary of the post and the bottom boundary of the microreactor is H, which denotes the nanogap height. The cross-sectional plane at $y = 0$ (**Figure S1, c and d**) further demonstrates the model geometry, including the location of the nanogap. This cross-sectional plane is also adopted as the geometry for the 2D simulation model.

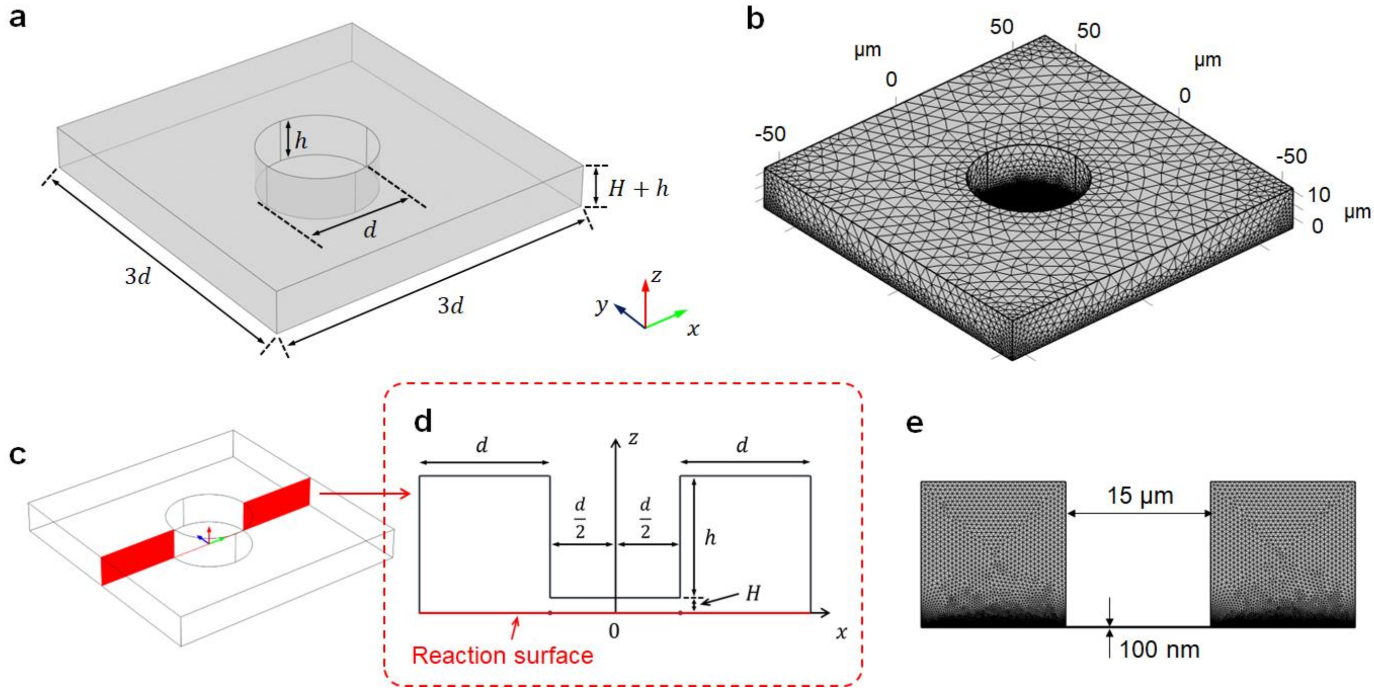

Figure S1. Simulation model of the micropost-induced perturbation of surface enzymatic reaction. (**a**) 3D simulation model of a microreactor. The depth, width, and height of the microreactor is $3d$, $3d$, and $H + h$. The coordinate origin is at the center of the bottom boundary of the microreactor. The height and diameter of the post is h and d. The nanogap height is H, which is the distance between the bottom boundary of the post and the bottom boundary of the microreactor. (**b**) An exemplary 3D model with a 40 µm post and a 100 nm thick nanogap showing the gradient structured mesh with the smallest mesh size and the highest mesh density at the surface under the post to appropriately capture the behaviors of the surface reaction and mass transfer in the nanogap. (**c**) The cross-sectional plane at $y = 0$ and (**d**) magnified diagram of the cross-sectional plane. The distance between the side boundaries of the post and microreactor is also d. (**e**) An exemplary 2D model with a 15 µm post and a 100 nm thick nanogap showing the gradient structured mesh used to appropriately capture the behaviors of the surface reaction and mass transfer in the nanogap.

Equations

The ALP/ELF-97 reaction as the model system to study the CHEMNLOCK process is described in **Fig. 3a**.

$$\mathrm{E} + \mathrm{S} \underset{k_2}{\overset{k_1}{\rightleftarrows}} \mathrm{E} \cdot \mathrm{S} \longrightarrow \mathrm{P}(aq) \longrightarrow \mathrm{P}(s)$$

.

In the simulation, we simplify the reaction model to a first-order reversible enzymatic reaction happening at the bottom boundary ($z = 0$) of the microreactor ($S \rightleftarrows P(aq)$) coupled to a first-order irreversible precipitation happening in the bulk of the microreactor ($P(aq) \rightarrow P(s)$)[1,2]:

$$\mathrm{S} \underset{k_2}{\overset{k_1}{\rightleftarrows}} \mathrm{P}(aq) \overset{k_p}{\longrightarrow} \mathrm{P}(s)$$

,

where k_1 and k_2 are the forward and reverse rate constants of the enzymatic reaction, respectively, and k_p is the rate constant of the precipitation reaction.

We denote the concentration of *S*, *P(aq)*, and *P(s)* as c_A, c_{P_1}, and c_{P_2}, respectively. The rate of the enzymatic reaction can then be described by

$$r_{forward} = k_1 c_A - k_2 c_{P_1}, \quad (1)$$

$$r_{reverse} = -k_1 c_A + k_2 c_{P_1}. \quad (2)$$

The precipitation reaction is described by a first-order irreversible kinetics model[2]. The reaction rate is proportional to the first-order kinetic reaction rate constant k_p and the difference between the solute concentration c_{P_1} and its saturation concentration c_{sat}[3,4].

$$r_{precipitation} = k_p\left(c_{P_1} - c_{sat}\right). \quad (3)$$

We introduce a control function:

$$\varphi(x) = \begin{cases} 0, x < 0 \\ 1, x > 0 \end{cases}. \quad (4)$$

Since the precipitation reaction will not happen until the solute concentration c_{P_1} reaches its saturation concentration c_{sat}, we couple the control function with Eq. (3) to describe the precipitation of *P(aq)* that happens in the bulk. This yields

$$r_{precipitation} = k_p\left(c_{P_1} - c_{sat}\right) \cdot \varphi\left(c_{P_1} - c_{sat}\right). \quad (5)$$

Since the fluid flow in the microreactor is stopped to prevent hydrodynamic disturbance of the spatial distribution of the enzymatic reaction products in the experimental design, convection is absent in all transport equations. The three species *S*, *P(aq)*, and *P(s)* have bulk diffusivity denoted by D_A, D_{P_1}, and D_{P_2}. All equations below are in dimensional form. The diffusion-reaction equations are constructed as follows[5–7]. Simulation constants can be found in **Table S1**.

For *S*, the governing transport equation in the bulk is

$$\frac{\partial c_A}{\partial t} = D_A \nabla^2 c_A. \quad (6)$$

The boundary condition for *S* at the reaction surface ($z = 0$) couples the enzymatic reaction rate:

$$\boldsymbol{n} \cdot (D_A \nabla c_A) = -k_1 c_A + k_2 c_{P_1}. \tag{7}$$

For all other boundaries, the boundary condition is no flux:

$$\boldsymbol{n} \cdot (D_A \nabla c_A) = 0. \tag{8}$$

S has an initial concentration of c_{A_0} in the bulk:

$$c_A = c_{A\_0}. \tag{9}$$

For *P(aq)*, the governing transport equation in the bulk is

$$\frac{\partial c_{P_1}}{\partial t} = D_{P_1} \nabla^2 c_{P_1} - k_p \left(c_{P_1} - c_{sat}\right) \cdot \varphi\left(c_{P_1} - c_{sat}\right). \tag{10}$$

The boundary condition for *P(aq)* at the reaction surface (*z* = 0) couples the enzymatic reaction rate:

$$\boldsymbol{n} \cdot \left(D_{P_1} \nabla c_{P_1}\right) = k_1 c_A - k_2 c_{P_1}. \tag{11}$$

For all other boundaries, the boundary condition is no flux:

$$\boldsymbol{n} \cdot \left(D_{P_1} \nabla c_{P_1}\right) = 0. \tag{12}$$

P(aq) has an initial concentration of $c_{P_1\_0}$ in the bulk:

$$c_{P_1} = c_{P_1\_0}. \tag{13}$$

For *P(s)*, the governing transport equation in the bulk is

$$\frac{\partial c_{P_2}}{\partial t} = D_{P_2} \nabla^2 c_{P_2} + k_p \left(c_{P_1} - c_{sat}\right) \cdot \varphi\left(c_{P_1} - c_{sat}\right). \tag{14}$$

The boundary condition for *P(s)* at all boundaries is no flux:

$$\boldsymbol{n} \cdot \left(D_{P_2} \nabla c_{P_2}\right) = 0. \tag{15}$$

P(s) has an initial concentration of $c_{P_2\_0}$ in the bulk:

$$c_{P_2} = c_{P_2\_0}. \tag{16}$$

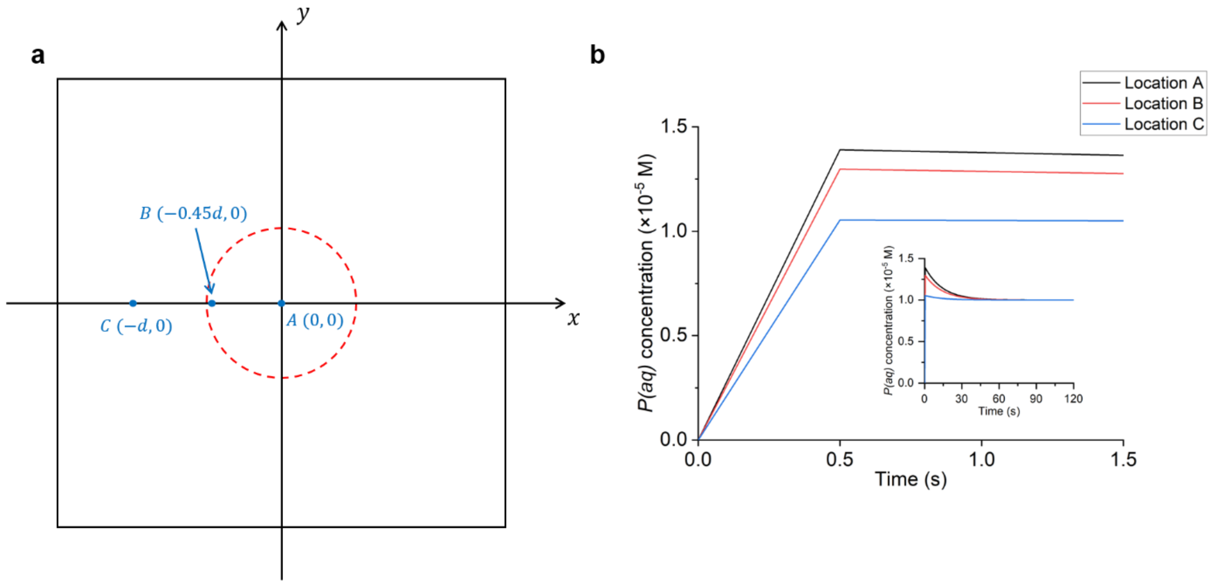

Figure S2. Nanogap confinement enhances the surface enzymatic reaction rate to reach the saturation level of *P(aq)*. (**a**) Three selected locations on the reaction surface (z = 0) to observe the effects of nanogap confinement on reaction rates. The coordinates of the three points are Location A (0, 0), Location B (-0.45d, 0), and Location C (-d, 0). The red dashed line indicates the location of the micropost projected onto the reaction surface. (**b**) Simulation results of the time evolution of *P(aq)* concentration at locations A, B, and C. Location A showed the highest reaction rate to reach the saturation level of *P(aq)* ($c_{sat} = 10^{-5}$ M), followed by Location B. Location C had the slowest reaction rate. Simulation model geometry: d = 15 μm and H = 100 nm.

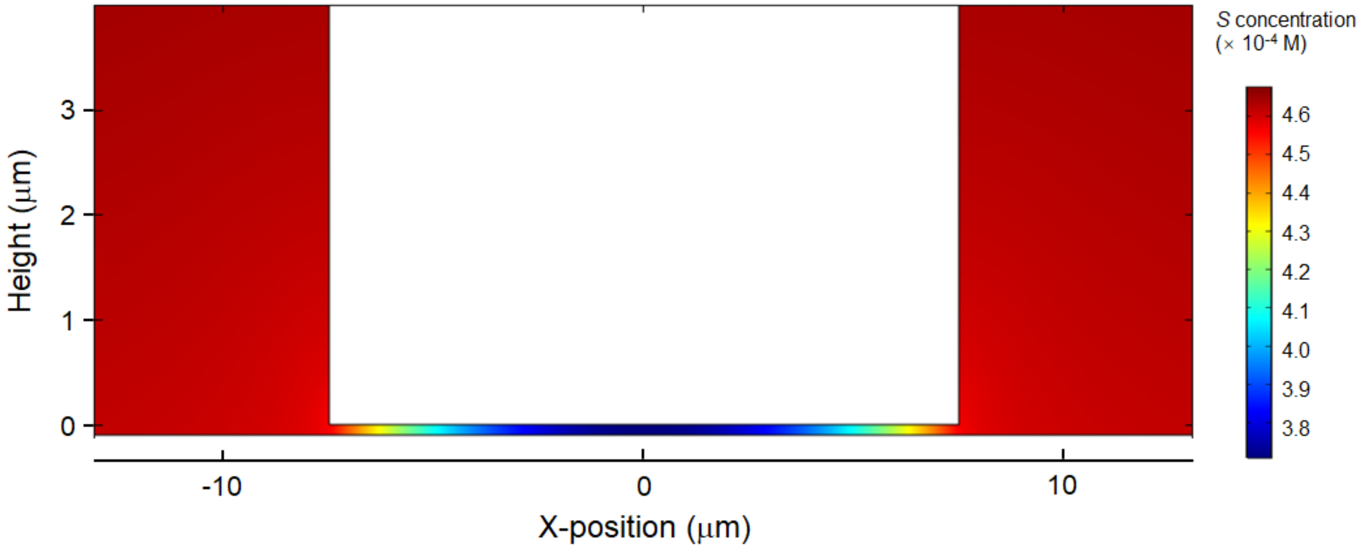

Figure S3. The concentration profile of *S* on the cross-sectional plane (*y* = 0) at *t* = 1 s. Color contour indicates the concentration magnitude. The concentration gradient of *S* from the bulk space to the nanogap is significantly larger than that across the open bottom surface. Simulation model geometry: d = 15 μm and H = 100 nm.

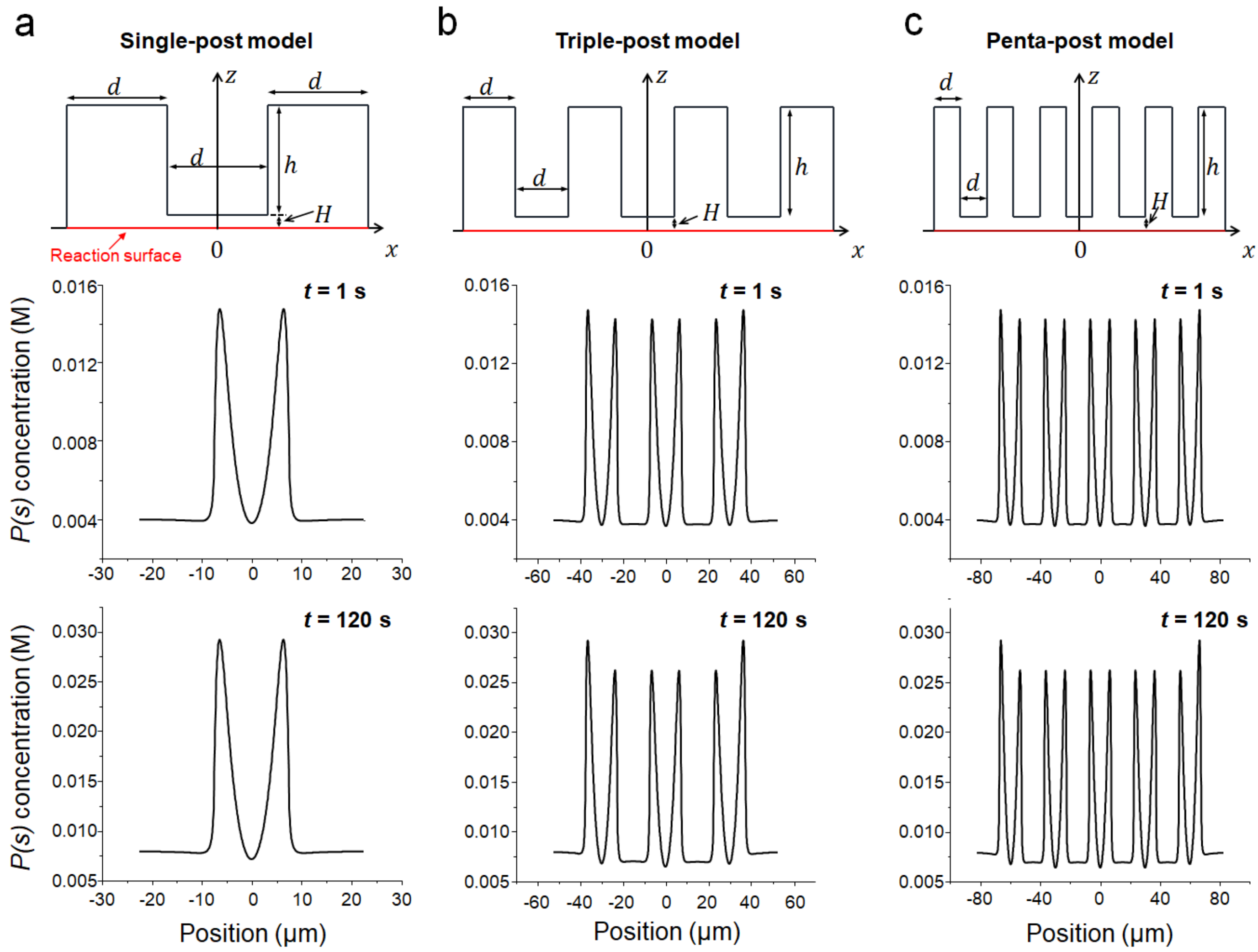

Figure S4. Multi-post systems yield consistent reaction modulation behavior compared with the single-post system. (**a-c**) The geometries of three 2D simulation models with one (**a**), three (**b**), and five (**c**) microposts (top panel) and the simulated *P(s)* concentration profile on the reaction surface obtained at two reaction time points, t = 1 s (middle) and t = 120 s (bottom) using these models. The post diameter, post interval, and the distance between the outermost post and the microreactor side boundary are all set as d. The side boundaries of the microreactor slightly affected the quantitative results of the *P(s)* concentration, but both the multi-post and single-post systems yielded consistent behavior with the most enhanced reaction at the gap entrance and suppressed reaction at the center. Simulation model geometry: d = 15 μm and H = 100 nm. Simulation rate constants: $k_1 = 10^{-5}$ m/s, $k_2 = 10^{-7}$ m/s, and $k_p = 10^3$ s^{-1}.

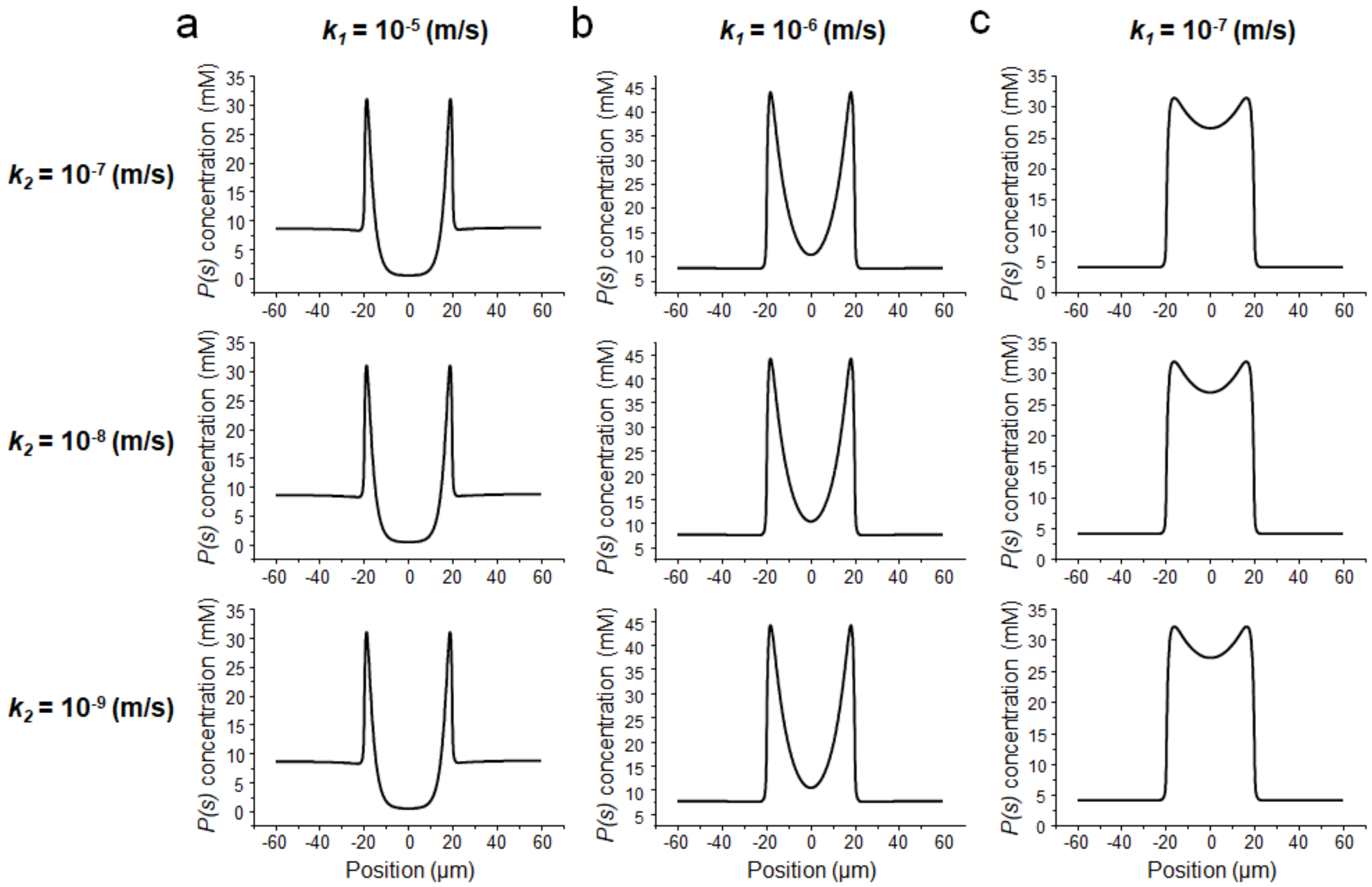

Figure S5. Assessment of the nanogap confinement effects by tuning surface reaction kinetics. (a, b, c) *P(s)* concentration profile on the reaction surface simulated with k_1 set to (**a**) 10^{-5} m/s, (**b**) 10^{-6} m/s, and (**c**) 10^{-7} m/s. Each k_1 value was assessed with k_2 decreased from 10^{-7} m/s to 10^{-8} m/s and to 10^{-9} m/s. Simulation model geometry: d = 40 μm and H = 100 nm. k_p is set as 1000 s^{-1} for all the cases.

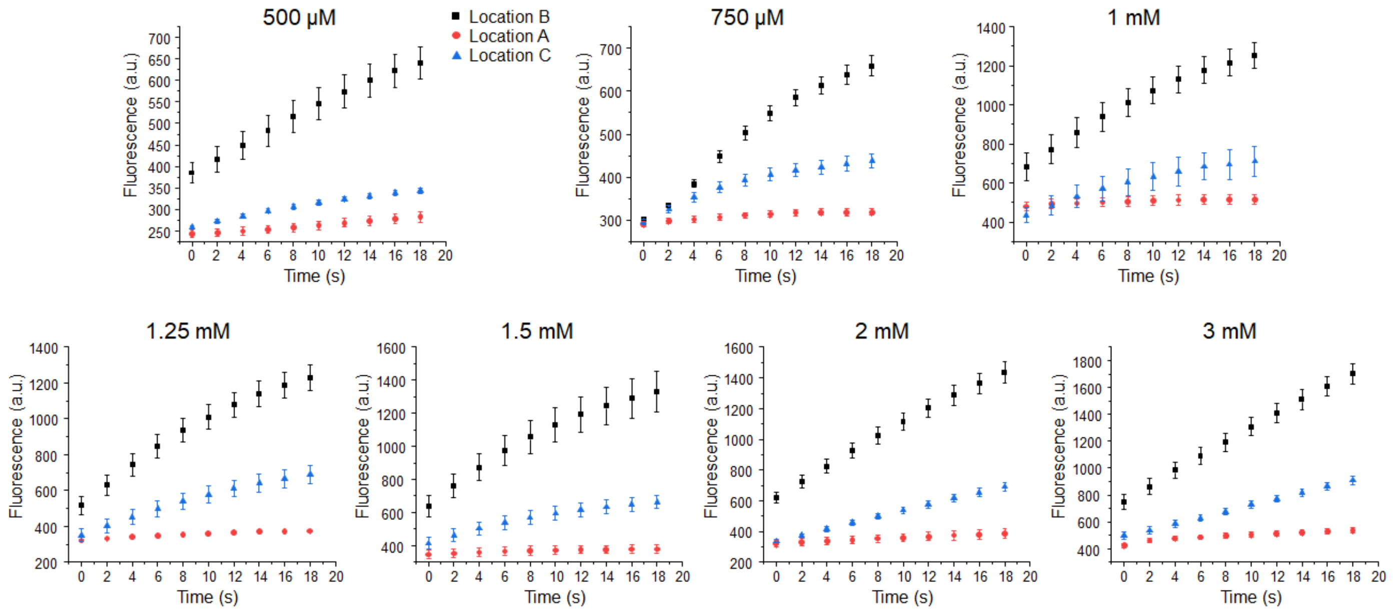

Figure S6. Time-lapse plots of the fluorescence intensity measured at the three designated locations using different ELF-97 concentrations. The steady-state assumption ($\frac{d[ES]}{dt} \approx 0$) for Michaelis–Menten model holds validity given the linearity of the rate of formation of fluorescent precipitates with respect to the time window of our measurement (the first five time points). Measured fluorescence intensity were presented as mean ± 1 S.D. ($n = 5$).

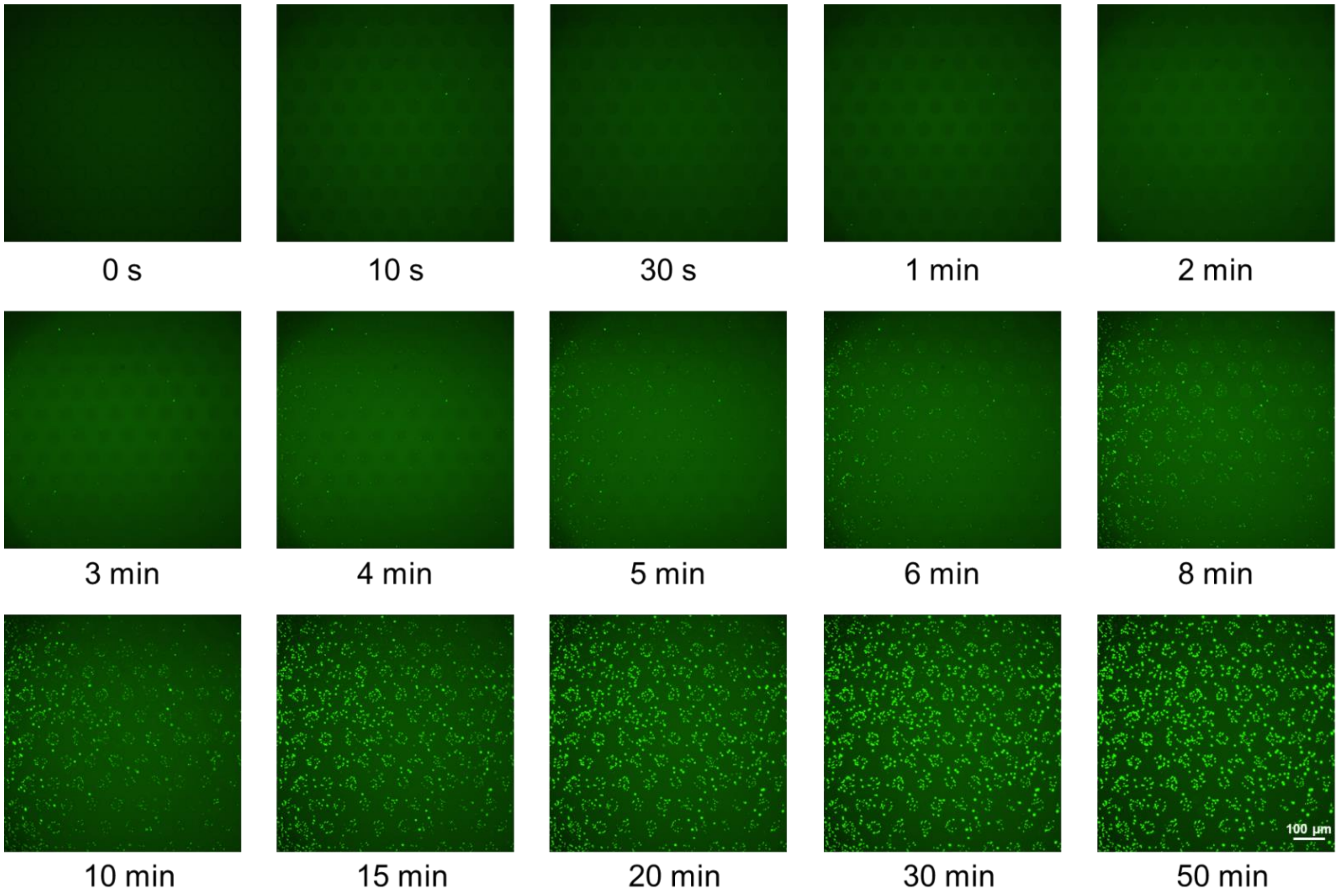

Figure S7. Time evolution of CHEMNLOCK-enhanced slow ALP/ELF-97 reaction. 250 μM ELF-97 and 0.6 μg/mL ALP were used. Enhanced formation of individual fluorescent dots was observed under microposts. The reaction became saturated after 20 min.

Table S1. Summary of simulation constants.

Notation	Description	Value
D_A	Diffusion coefficient of ELF-97[a]	6.3×10^{-10} m^2/s
D_{P_1}	Diffusion coefficient of dissolved ELF-97 alcohol[a]	7.0×10^{-10} m^2/s
D_{P_2}	Diffusion coefficient of precipitated ELF-97 alcohol[b]	0 m^2/s
c_{sat}	Saturate concentration of dissolved ELF-97 alcohol[c]	10^{-5} M
c_{A_0}	Initial concentration of ELF-97	500 μM

[a] Diffusion coefficients of ELF-97 and dissolved ELF-97 alcohol molecules in PBS were estimated using Wilke-Chang correlation equations[8].

[b] The precipitated product is assumed to be stationary.

[c] See reference[9].

Table S2. Summary of the apparent Michaelis-Menten parameters for the enzymatic studies.

	**Location A**	**Location B**	**Location C**
V_{max}^{app} (a.u./s)	6.85 ± 1.34	86.74 ± 7.44	46.98 ± 5.02
K_M^{app} (mM)	1.31 ± 0.48	1.47 ± 0.25	2.60 ± 0.42

References

1. Murray, J. D. Reaction Kinetics. in *Mathematical Biology* (ed. Murray, J. D.) 109–139 (Springer, Berlin, Heidelberg, 1993). doi:10.1007/978-3-662-08542-4_5.
2. Dijk, P. & Berkowitz, B. Precipitation and dissolution of reactive solute in fracture. *Water Resources Research - WATER RESOUR RES* **34**, 457–470 (1998).
3. Bird, G., Boon, J. & Stone, T. Silica transport during steam injection into oil sands: 1. Dissolution and precipitation kinetics of quartz: New results and review of existing data. *Chemical Geology* **54**, 69–80 (1986).
4. Weir, G. J. & White, S. P. Surface deposition from fluid flow in a porous medium. *Transp Porous Med* **25**, 79–96 (1996).
5. Ramachandran, P. A. *Advanced Transport Phenomena: Analysis, Modeling and Computation*. (Cambridge University Press, 2014).
6. Deen, W. M. *Analysis of Transport Phenomena*. (Oxford University Press, 2012).
7. Grodzinsky, A. J. *Fields, Forces, and Flows in Biological Systems*. (Garland Science, 2011).
8. Wilke, C. R. & Chang, P. Correlation of diffusion coefficients in dilute solutions. *AIChE Journal* **1**, 264–270 (1955).
9. Huang, Z., Terpetschnig, E., You, W. & Haugland, R. P. 2-(2′-Phosphoryloxyphenyl)-4(3H)-quinazolinone derivatives as fluorogenic precipitating substrates of phosphatases. *Analytical Biochemistry* **207**, 32–39 (1992).
